# Growth and Mechanical Characterization of Mycelium-Based Composites towards Future Bioremediation and Food Production in the Material Manufacturing Cycle

**DOI:** 10.3390/biomimetics7030103

**Published:** 2022-07-28

**Authors:** Thibaut Houette, Christopher Maurer, Remik Niewiarowski, Petra Gruber

**Affiliations:** 1 Department of Biology, The University of Akron, Akron, OH 44325, USA; rdn19@uakron.edu; 2 Redhouse Studio, Cleveland, OH 44113, USA; chris@redhousestudio.net; 3 Transarch Office for Biomimetics and Transdisciplinary Architecture, 3370 Ybbs an der Donau, Austria; peg@transarch.org

**Keywords:** mycelium, fungal architecture, myceliated material, living material, sustainability, biotechnology, compression, bending, waste upcycling, mycoremediation

## Abstract

Today’s architectural and agricultural practices negatively impact the planet. Mycelium-based composites are widely researched with the aim of producing sustainable building materials by upcycling organic byproducts. To go further, this study analyzed the growth process and tested the mechanical behavior of composite materials grown from fungal species used in bioremediation. Agricultural waste containing high levels of fertilizers serves as the substrate for mycelium growth to reduce chemical dispersal in the environment. Compression and three-point bending tests were conducted to evaluate the effects of the following variables on the mechanical behavior of mycelium-based materials: substrate particle size (with or without micro-particles), fungal species (*Pleurotus ostreatus* and *Coprinus comatus*), and post-growth treatment (dried, baked, compacted then dried, and compacted then baked). Overall, the density of the material positively correlated with its Young’s and elastic moduli, showing higher moduli for composites made from substrate with micro-particles and for compacted composites. Compacted then baked composites grown on the substrate with micro-particles provided the highest elastic moduli in compression and flexural testing. In conclusion, this study provides valuable insight into the selection of substrate particle size, fungal species, and post-growth treatment for various applications with a focus on material manufacturing, food production, and bioremediation.

## 1. Introduction

### 1.1. Problem Statement of Current Building Materials

Today’s architectural practices negatively impact the planet’s ecosystems. The building industry and its energy-intensive material manufacturing processes were responsible for a total of 37% of global carbon emissions in 2020, and these processes contribute significantly to anthropogenic climate change and resulting extreme weather events [1]. The push for sustainability in architecture needs to encompass the entire life cycle of materials and buildings, from resource extraction to repurposing or disposal. Even simple materials used in the building industry are becoming scarce and limited. At the other end of the building process, 600 million tons of construction and demolition waste were generated in 2018, with 145 million tons sent to landfills [2]. As a result, landfilled hazardous materials, such as lead, can contaminate ground water [3]. Even after waste removal, sites contaminated with heavy metals and toxic chemicals from industry still need to be cleaned to limit run-off and spread to the environment. Therefore, the building industry urgently needs to focus on cleaner materials that can be repurposed, to reduce both material scarcity and waste generation.

### 1.2. Towards Living Materials

In response to the current limitations of traditional architectural materials and their manufacturing practices, living materials (i.e., materials integrating biological organisms) have emerged in recent decades. Specifically, Engineered Living Materials (ELMs) are defined as genetically or mechanically “engineered materials composed of living cells that form or assemble the material itself, or modulate the functional performance of the material in some manner” [4]. The implementation of living organisms in technological materials allows engineers to benefit from the qualities of biological growth [5]. The overall advantages of ELMs are self-production, clean chemistry, sustainability, adaptability, self-healing, and the potential for added functionality through genetic and mechanical engineering. The wide scope, potential, and limitations of ELMs have been discussed in multiple reviews [4,6]. Examples of ELMs include microbially manufactured polymer matrices, soft living robots, smart living surfaces, living carbon composites, bacteria-based self-healing concrete, bacterial cellulose, biologically fabricated bricks, and mycelium-based materials. The long-term goal of ELMs is to build large-scale hierarchical material systems from simple autonomous micro-entities in situ. However, more research needs to be conducted to keep organisms alive in the applied setting, scale up production of a laboratory environment, and predict organisms’ behavior. 

### 1.3. Mycelium-Based Materials

Fungi are used for ELMs because of their mycelium, which forms a 3D binding network, secretion of enzymes, diversity of properties between species, and wide range of material applications. Mycelium-based materials are among the most successful large-scale living materials [4,7]. Mycelium-based materials are produced by growing fungal mycelium on an organic substrate (e.g., often agricultural byproducts) in a mold. A variety of post-growth treatments are applied based on the desired application. The material properties are highly tunable based on the selected substrate type (i.e., chemically, and related to size), fungal species, growth environment, and post-growth treatments [5,7,8,9,10,11,12,13]. The ability to fine tune the properties of mycelium-based materials increases their range of application, including packaging, electronics, acoustic absorbers, footwear, insulators, fire protection, and self-healing materials [8,14,15,16,17,18,19,20,21,22,23]. At the end of their life cycle these composite materials are biodegradable and can even be used as a substrate for growing new iterations of materials. Therefore, mycelium-based materials promote organic waste upcycling, low-energy material manufacturing, and biodegradable materials, making them an alternative to current architectural materials. To showcase their architectural potential, temporary installations have been built with mycelium-based materials [5,24,25,26,27,28,29]. To address the limitations of ELMs, more studies need to be conducted before their implementation in permanent buildings by exploring new substrate/fungus combinations, evaluating the effect of various parameters (e.g., fungal species, substrate type and size, growth environment, and post-growth treatments) on material properties, predicting material behavior, ensuring homogeneous material properties, and characterizing the accumulation and decomposition of toxic chemicals [5].

### 1.4. State of the Art of the Production and Mechanical Properties of Mycelium-Based Materials 

Throughout the production process of myceliated materials, various factors influence their acoustic, thermal, mechanical, and physical characteristics [12,13]. Different fungal species will feed on different substrates and grow at different rates [30]. The mycelium anatomy and structure differ between species, with three main categories found in basidiomycetes: monomitic (i.e., generative), dimitic (i.e., generative, and skeletal), and trimitic (i.e., generative, binding, and skeletal) [13]. For example, myceliated materials grown from trimitic species (e.g., *Trametes versicolor*) display a higher compressive, tensile, and flexural strength than those grown from monomitic species (e.g., *Pleurotus ostreatus*) [31,32]. The substrate forms the base of the composite materials as it is not completely decomposed by the fungus during the mycelial growth process. Therefore, the substrate composition and particle size affect the material properties of the end material as it serves as its backbone structure [13,33]. To successfully introduce the desired fungus, the substrate must be cleaned of other species. By removing competing species, this fungus can grow over the entire substrate and produce a coherent material. Different techniques are used to kill competing species: sterilization (e.g., autoclaving, bathing in hydrogen peroxide, bathing in a basic solution, baking) or pasteurization (e.g., steaming) [5,13]. These techniques affect the mycelial growth speed and removal of competing species differently. 

During inoculation, the proportions of the ingredients (e.g., dry substrate, water, mycelium spawn, nutrients) for optimal mycelial growth vary between fungal species. As mycelial growth highly depends on the nutrient profile of the substrate, food with high nutritious content is often added to the agricultural byproducts. Since mechanical failure always occurs in the mycelium binder, mycelial growth and, therefore, the nutrient profile of the substrate, are especially important for mechanical performance [13]. However, the use of more-nutritious substrate makes the material less impactful in terms of sustainability, waste upcycling, and in general, resource efficiency. Percentages of ingredients used in previous research projects are shown in Appendix A. 

The growth environment (i.e., temperature, relative humidity, access to oxygen, clean/ventilated air exchange, and lighting conditions) also impacts mycelium growth. The temperature should be kept at around 25–30 °C [7,13,34] and the relative humidity around 70–80% [7]. Based on the fungal species, substrate, growth environment, and level of growth desired, the growth time may vary from 6 days [35] to 20 days [7], and up to months [13,34]. In the literature, the growth of fruiting bodies in the production of mycelium-based materials is avoided as it can consume resources otherwise used for mycelium growth, modify material shape, increase composite heterogeneity, require maintenance (i.e., harvest required in an environment that should remain as clean and undisturbed as possible to avoid contamination), and release spores that could cause allergic reactions or infections [32,36,37,38,39,40,41]. Fruiting body formation can be inhibited by controlling both the lighting conditions and carbon dioxide concentration (i.e., dark with low CO_2_) or by introducing GSK-3 inhibitors [36,37]. During the growth process and the drying period, myceliated materials shrink. Knowing the shrinkage of the material is important to estimate and target specific product dimensions. For cylindrical specimens, the shrinkage has been estimated to be around 17% in height (vertical shrinkage) and around 10% in diameter (horizontal shrinkage) [9]. For rectangular specimens, shrinkages of 5.56% horizontally and 2.78% vertically have also been observed [42].

After the growth process, various treatments (e.g., drying, baking, compacting, coating) can be applied to the myceliated materials to tune their properties [13]. Existing studies do not usually differentiate drying and baking of the specimens. Furthermore, there is no standard practice for drying or baking, as their success depends on specimens’ dimensions. For example, various research teams used the following drying/baking techniques: 40 °C for 72 h plus 2 h at 100 °C [33], 60 °C for 24 h [8], 60 °C for 2 h [7], 70 °C for 5 to 10 h until the weight is stabilized [9], 80 °C up to a constant weight [21], 80 °C for 24 h [32], 100 °C for 4 h [35,43], and 100 °C for several hours [44]. The mycelial structure is believed to degrade with temperatures of approximatively 225 to 300 °C [7].

Various research articles studied the mechanical properties of mycelium-based materials, which have been described by a two-phase particulate model with the mycelium as the matrix and substrate as the dispersed phase [7,8,9,10,11,42]. The differences in the production, dimensions, description, and testing procedures of such bio-composite materials make data comparison between studies difficult, as they severely impact their mechanical behavior [42]. The mechanical behavior of mycelium-based materials highly depends on the anisotropic substrate matrix. A study recently looked at the effect of fiber orientation within the substrate on the compressive behavior of mycelium-based materials [42]. The study found that adding fibers oriented in the direction of loading increased Young’s modulus. Conversely, fibers oriented perpendicular to the loading direction produced a decrease in Young’s modulus and ultimate strength. A study found that myceliated materials made from loose substrate had lower compressive Young’s moduli than those from chopped substrates [9]. The same study also found variability in compressive Young’s moduli based on substrate used, despite their similar density (around 100 kg/m^3^): from 0.14 MPa for loose pine shavings samples up to around 1.25 MPa for pre-compacted hemp and flax samples. Myceliated samples grown with *Pleurotus ostreatus* on hemp mat had a compressive strength of 0.19 MPa compared to 0.26 MPa for *Trametes versicolor* [31]. Another study found a compressive modulus of 1.3 MPa for *Ganoderma lucidum* grown on macerated red oak wood chips (5–15 mm) and a nutrient solution with a final density of 318 kg/m^3^ [45]. Vidholdová et al., 2019, grew low-density mycelial boards having a density of 103 kg/m^3^, which resulted in a compressive resistance at 20% strain of 23.95 kPa [11]. Compressive performance of porous materials increases with increasing density, which may result from a variety of parameters including the substrate used, its particle size, the degree of compaction, and the amount of substrate digested by the fungus [13,46,47]. Islam et al., 2017, studied the correlation between density and uniaxial elastic modulus on mycelium boards from Ecovative Design LLC (Green Island, NY, USA) [48]. They found that densities in the range of around 150 to 160 kg/m^3^ lead to elastic moduli in the range of 0.5 MPa to 1.1 MPa, respectively. Yang et al., 2017 found various Young’s moduli ranging from around 5 to 50 MPa for densities ranging from around 160 to 280 kg/m^3^ made with *Irpex lacteus* mycelium grown on a variety of substrates including wood pulp, millet grain, wheat bran, natural fiber, and calcium sulfate [8]. In terms of bending properties, a study looked at the mechanical properties of bioresin-infused mycelium-based sandwich composite materials under 3 pt bending, which led to an elastic modulus of 1.13 MPa for a density of 121.7 kg/m^3^ [49]. Appels et al., 2019, found an increase in flexural moduli from non-pressed (ranging from 1 to 9 MPa for densities from 100 to 170 kg/m^3^) to cold-pressed (ranging from 12 to 15 MPa for a mean density of 240 kg/m^3^) and hot-pressed (ranging from 34 to 80 MPa for densities from 350 to 390 kg/m^3^) in materials grown from *Trametes multicolor* or *Pleurotus ostreatus* on rapeseed straw, beech sawdust, or cotton [32]. Further, Appels et al., 2018, found that controlling the lighting conditions and carbon dioxide levels of the growth environment (i.e., light with CO_2_ content), in addition to deleting the hydrophobin gene *sc3*, increased composite density and resulting Young’s modulus [38]. 

Due to the novelty of the research field of mycelium-based composites, many research questions still need to be addressed before their implementation in permanent architectural projects [5]. For instance, more studies should explore particle shapes, composition, and distributions due to their significant influence on mechanical properties [42]. The substrate particle size for an optimal balance between mycelial growth and mechanical performances is still subject to research. Higher substrate density lowers air transmission, resulting in limited mycelial growth inside the substrate if the substrate is not artificially aerated [13]. However, Islam et al., 2018, found that the generic trends of the stress–strain curves from compressing mycelial composite made of different particle sizes were not sensitive to the particle size, suggesting that the myceliated materials’ compressive response is independent of substrate particle size [43]. In their study, they compared five different substrate particle sizes with varying aspect ratios (2, 5, or 8), sizes (2.5, 5, or 10 mm), and diameters (0.5, 1, or 2 mm). In another study, materials made from medium particles (0.75–3.0 mm) led to higher density, Young’s modulus, and ultimate strength than others made from smaller (0.5–1.0 mm), larger (4.0–12.0 mm), or more diverse (0.5–12.0 mm) particles [42]. This study also showed that higher density did not increase compressive performance in all cases. To the author’s knowledge, no study has questioned the effect of the very fine micro-particles on mycelial growth speed and mechanical performance. Moreover, the substrate particle size will likely influence the effects of different post-growth treatments. To further extend the analysis of substrate size, more studies should evaluate the subsequent effects of post-growth treatments on composites grown with these various substrate sizes. As a result, the combined effects of mutually responsive variables need to be addressed in future research. 

### 1.5. Underutilized Benefits of Fungal Mycelium 

The variety of fungal species possess many qualities unexploited in current mycelium-based materials. Fungi are known for their nutritional, medicinal, and bioremediation benefits [50,51]. Fungi release enzymes to break down substances that they can feed upon. This behavior makes them very interesting for mycofiltration (i.e., use of fungi to filter water) and mycoremediation (i.e., use of fungi to decontaminate/depollute the environment). Whereas mycofiltration relates to filtering water, mycoremediation aims at decontaminating a substrate. Mycoremediation is a subset of bioremediation practices, in which fungi serve to uptake and break down pollutants. It has been referenced as the cheapest remediation technique for polycyclic aromatic hydrocarbons, at approximately 50 USD/ton [51]. Paul Stamets described a circular model in which fungi serve to produce food and medicine, remediate soil, and facilitate plant growth [51]. Thanks to recent mycelium-based materials research, the production of building materials can now be integrated in this model by growing mycelium materials on contaminated substrate and/or harvesting fruiting bodies during the growth process. Depending on the type of contamination and fungal species employed, toxins present in the substrate are decomposed by the fungus, stored in the mycelium, or accumulated in the fruiting bodies, which are harvested for treatment. In all cases, the myceliated substrate can serve for producing composite materials once their toxicity is evaluated. As stated in Section 1.4, the growth of fruiting bodies is currently avoided in the production of composite materials for various reasons. In terms of mechanical performance, the authors did not find any study comparing the mechanical behavior of mycelium-based materials grown with and without fruiting bodies. Therefore, a study should be conducted to validate the hypothesis that fruiting body formation lowers mechanical performance of these materials. Depending on the targeted application, the authors believe that the benefits of producing fruiting bodies for food or medicinal applications outweigh the potential reduction in mechanical performance. For instance, fruiting bodies could be harvested during the mycelium growth of composite materials. Specific frames should be used to allow harvest without disturbing the growth environment and introducing contaminants. Another solution is to upcycle the fruiting block serving for mushroom production after it is spent, by using it as myceliated substrate for material production [52]. In conclusion, more studies should be performed to evaluate the potential integration of material production in the circular model of current utilizations of fungi.

### 1.6. Overall Goal of the Project

A potential for this updated model is to grow bioremediating species on contaminated organic substrate from sources including the agriculture and building industries. In addition to decontaminating the substrate, food (i.e., fruiting bodies) and materials (i.e., mycelium-based composites) can be produced after toxicity evaluation. Depending on the chemicals used to accumulate or decompose and the desired application, various bioremediating fungal species can be employed. For instance, *Pleurotus ostreatus* (commonly named oyster mushroom) is a recommended species to decontaminate petroleum products [51,53]. Another species often seen in polluted soils, *Coprinus comatus* (commonly named shaggy mane), is a bio-accumulator of heavy metals and a species recommended for decontamination of substrates with nitrates and phosphorus-bound toxins [51]. Therefore, specific fungal species can be used depending on the chemicals present in the substrate. During mycelial growth over the enriched substrate, fruiting bodies are harvested for chemical treatment or food based on their toxicity. Upon full mycelial coverage, the composite material receives various treatments to tune its properties. Finally, the mycelium-based material and fruiting bodies produced would be chemically tested to ensure that they are safe for the desired application. 

This article presents a first step towards the implementation of this model in a case-study. Two bioremediating species (*Coprinus comatus* and *Pleurotus ostreatus*) were grown on an agricultural byproduct (straw) to produce composite materials. The mycelium-based materials produced were mechanically characterized through compression and bending testing to assess their potential for architectural purposes. These tests sought to evaluate the effects of the following variables on composite mechanical behavior: substrate size (with or without micro-particles), fungal species (*Coprinus comatus* or *Pleurotus ostreatus*), and post-growth treatment (dried, baked, compacted then dried, compacted then baked). 

To complete the model validation, chemical tests would need to be performed in a further study. These tests were not generated in this part of the project due to a cut in resources (expertise and money) and delays emerging from COVID-19 regulations. However, all mycelium-based materials and samples of both substrate mixtures were stored to be used in future chemical analysis.

## 2. Materials and Methods

**Figure 1:**
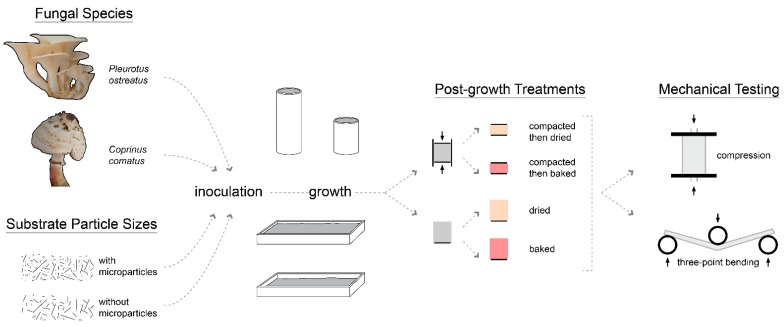
This figure shows the overall process of the project, including the variables evaluated (i.e., fungal species, substrate particle size, and post-growth treatment) for their effect on the mechanical properties (i.e., under compression and bending) of the composite material.

The main steps of the project and variables studied are illustrated in Figure 1. 

### 2.1. Substrate and Fungal Species

The substrate used for mycelium growth was straw, collected in bales from Dussel Farm, Ohio, USA. Different batches of mycelium-based materials were produced with two different fungal species (*Pleurotus ostreatus* and *Coprinus comatus*). Due to limited availability of resources and the scale of the project, the batches were not conducted at the same time. Mycelium-based materials grown with *Pleurotus ostreatus* were manufactured from January to June 2020, while those with *Coprinus comatus* were produced from June to September 2020. The same process was used for both batches: preparation of the straw substrate, inoculation, mycelium growth, application of post-growth treatments, and mechanical testing.

### 2.2. Substrate and Mold Preparation

#### 2.2.1. Chipping

To reduce the size of the substrate particles, the straw was chipped (Done Right Chipper Shredder Premier 300, Generac Power Systems, Inc., Waukesha, WI, USA). Chipping straw increases substrate density, as a reduction in particle dimension increases packing density. The density of the straw increased from 121.69 kg/m^3^ before chipping to 177.28 kg/m^3^ after. The chipper produced particles with varying lengths, including micro-particles. Due to the dispersal of particles into the environment, 17.93% and 21.62% of the straw weight was lost during the chipping process for the first and second batch, respectively. Both batches were chipped at different times of the year (January and June). The difference in straw weight loss likely results from the divergent environmental conditions (i.e., mostly humidity and wind) during chipping.

#### 2.2.2. Sieving

Since the chipper produced particles of variable dimensions, the chipped particles were sieved to control the particle size. Two different particle size sets were produced for this research project to compare their effect on mycelial growth speed and mechanical performance. The first set of particles (S for small) was chipped and sieved through a 5.7 mm sieve to exclude large particles. The second set of particles (L for large) was similarly chipped and sieved through a 5.7 mm sieve. In addition, the particles were then sieved through a 1.5 mm sieve; only the material stuck on the sieve was kept and fine particles were removed. In conclusion, fine particles (passing through a 1.5 mm sieve) were kept in mixture S, while they were removed from mixture L. The two mixtures of particles (L and S) served as substrate to assess the effect of the fine particles on the mycelial growth and mechanical performance. The meshes of the 5.7 mm sieve (0.22-inch opening) and 1.5 mm sieve (0.06-inch opening) are respectively closest to standard US meshes No. 30 and No. 14. Additional details on sieving non-spherical objects and the sieving procedure are given in Appendix B.

For the first batch, the sieving process only resulted in 1.05% weight loss for the 5.7 mm sieve, and 0.51% for the 1.5 mm sieve. After the chipping and sieving processes of the first batch, 80.57% of the unchipped straw weight (100%) remained as the small mixture. Due to the removal of fine particles, only 42.73% of the unchipped straw weight remained as the large mixture. For the second batch, 66.13% and 38.52% of the unchipped straw weight resulted in the small and large mixtures, respectively. Both batches were sieved by a different individual, which may be the cause of the different percentages observed despite following the same procedure.

#### 2.2.3. Quantifying Particle Sizes

As stated in Section 2.2.2., the dimensions of the particles passing through a sieve may be larger than the opening size of the sieve. Therefore, a technique was developed to quantify the mean size of the particles of mixtures S and L. The same chipping and sieving processes were performed on a known quantity of straw to produce both mixtures. Particles from each sieving step were then measured by placing them on a light table; a picture of them was taken and their 2D dimensions were extracted using an image analysis algorithm. This process is described in detail in Appendix C. For each set of particle sizes, this entire process was repeated five times with new particles from the same mixture. 

#### 2.2.4. Sterilization and Pasteurization

Due to inaccessibility of specific resources (e.g., pasteurization and inoculation equipment and workforce) under COVID-19 regulations, the first batch of substrate was sterilized and the second was pasteurized. For the first batch, the dry chipped and sieved substrate was sterilized in 10 autoclaving bags weighing around 2422 ± 109 g for mixture L and 1918 ± 152 g for mixture S. Each bag was sterilized for 30 min at 121 °C and 16 psi inside an autoclave at the Biology Department of The University of Akron. Autoclaved bags remained sealed until inoculation to reduce the entry of contaminants. For the second batch, both mixtures were pasteurized one after the other at Valley City Fungi, OH, USA. Mixture L was pasteurized first to avoid the transfer of fine particles from mixture S to L. The pasteurization process is described in Appendix D. Both mixtures were finally placed into sealed bags to avoid contamination in transport and following manipulations.

#### 2.2.5. Mold Preparation

The molds into which the inoculated substrate was placed for mycelium growth were built based on the ASTM testing requirements. Due to the absence of ASTM standards specific to mycelium-based materials, various related standards had to be analyzed. Based on their similarity with mycelium-based physical properties and previous research studies, ASTM D2166/D2166M-13 was used for compression testing and ASTM D1037-12 for bending testing [8,9,54,55]. According to these standards, cylindrical molds (i.e., PVC pipes) were built for specimens to be tested in compression and rectangular molds (i.e., wooden frames) for those to be tested in bending.

For compression testing, the targeted dimensions were 7 cm in diameter and 14 cm in height (i.e., height-to-diameter ratio of 2:1) as described in ASTM D2166/D2166M-13 [55]. However, it was anticipated that specimens would shrink during the drying process. To anticipate shrinkage and test specimens following the dimensions specified by the ASTM standard, specimens were made 8% larger in diameter and 20% larger in height based on estimations from the literature and preliminary experiments. Furthermore, it was expected that half of the specimens would be compacted to half of their height after the growth period (post-growth treatment detailed in Section 2.4). In this regard, specimens were grown inside 3 in. × 10 ft. PVC tubes (Charlotte PVC 40 Plain-End DWV Pipes, Charlotte Pipe and Foundry, Charlotte, NC, USA), with a diameter of 7.7 cm and a height of 17 or 34 cm. 

For the bending test in ASTM D1037-12, materials thicker than 0.6 cm should have a width of 7.6 ± 0.1 cm and a length of (5.1 cm + 24 × the nominal thickness) [54]. Bending tests were performed with an Instron 5567 electrochemical testing system (Instron, Norwood, MA, USA), which can fit specimens up to a length of around 35.56 cm. Therefore, the specimens’ dimensions were selected to be as follows: a length of 32.66 cm, a width of 7.60 cm, and a thickness of 1.15 cm. The same shrinkage percentages as per compression testing specimens were used to produce specimens 8% larger in width and length and 20% thicker. To optimize space and material, each mold was built to contain six specimens side by side. Half of the specimens were also grown twice as thick to prepare for compaction to half of their thickness. These molds were made of ½ in. medium-density fiberboard (MDF) boards (Home Depot, Atlanta, GA, USA) to grow a large mycelium-based specimen measuring 35.5 cm in width, 50 cm in length, and 1.4 or 2.8 cm in thickness. A layer of plastic made from polyethylene resin (Home Depot, USA) was laid inside the mold to keep the mycelium from growing on and sticking to the wooden mold. Additional rectangular molds measuring 49.2 cm in width, 55 cm in length, and 1.4 or 2.8 cm in thickness were produced to grow leftover material. 

### 2.3. Inoculation

Using existing research as a baseline (see Appendix A) and discussions with mushroom farmer John Burmeister from Valley City Fungi, Ohio, USA, the team decided on a recipe consisting of 10 wt% *Pleurotus ostreatus* or *Coprinus comatus* grain spawn, 22.5 wt% dry substrate (chipped and sieved straw), and 67.5 wt% water for both batches. The first batch was inoculated in redhouse studio’s warehouse in Cleveland, Ohio, USA, where all materials were grown. For the second batch, the inoculation of both mixtures was performed immediately after pasteurization in the same container at Valley City Fungi, OH, USA. The inoculation process of both batches and a summary of the quantity of specimens grown are detailed in Appendix E. 

### 2.4. Mycelium Growth

To allow for comparison between fungal species, the growth environments and growth time were kept the same for both batches. The mycelium-based materials were grown for 6.5 weeks (i.e., 46 days) in a dark opaque tent. The temperature and relative humidity inside the tent were 25.7 ± 0.2 °C and 50.5 ± 5.6%, respectively, during the growth period. However, each specimen was grown inside a closed mold and then placed inside the large tent. The relative humidity inside each closed mold was based on the water concentration of the mycelium mixture and was considered to have remained constant throughout the growth period, as each mold was sealed with non-woven housewrap. Batches were grown at a different time of the year: from 21 March to 6 May 2020 (first batch) and from 29 June to 14 August 2020 (second batch). Mycelial growth and presence of contaminants were visually assessed throughout the growth period through the clear plastic for both batches. After 6.5 weeks of growth, all the non-woven housewrap material covering the top of the molds was removed to start the drying process of the specimens. Additional details on the growth data collected and removal of contaminated parts are explained in Appendix F. 

### 2.5. Application of Post-Growth Treatments

Grown materials received were either dried, baked, compacted then dried, or compacted then baked. Drying myceliated materials dehydrates them to make the fungus dormant and stop its growth. If the water content of the materials is increased after the drying process, the fungus is usually able to resume growth [5]. Myceliated materials were dried in an oven for a total of 12 h at 40 °C. Due to the restrictions of the COVID-19 regulations, they were dried in 2 sessions of 6 h each and kept in the oven between these sessions.

The baking process is supposed to kill the fungus. In comparison with the drying process, if the water content of the material is increased after the baking process, no fungal growth should be observed. They were placed in an oven for 6 h at 100 °C to bake.

Half of the specimens were compacted to half of their thickness with an industrial hydraulic press (20 TON shop press from Central Machinery, Moses Lake, WA, USA) between metal plates. Further details on the application of post-growth treatments are explained in Appendix G.

### 2.6. Mechanical Testing Procedure

#### 2.6.1. Compression Testing

Due to the lack of testing standards to assess the uniaxial compressive properties of myceliated materials, various ASTM standards specific to soil (i.e., ASTM D2166/D2166M-13), wood-based panels (i.e., ASTM D3501 and ASTM D1037), and thermal insulations (i.e., ASTM C165-07) have been used by research teams [8,9,11,42,55,56,57]. Elsacker et al., 2019, followed ASTM D3501, whereas their use of cylindrical specimens followed ASTM D2166/D2166M-13 [9]. This latter standard specifies that the specimen should have a height:diameter ratio ranging from 2:1 to 2.5:1. In their study, Elsacker et al. opted for a 0.5:1 height:diameter ratio with specimens measuring 3.75 cm in height (or 10 cm in height for pre-compressed specimens) for 7.5 cm in diameter [9]. Yang et al., 2017, followed ASTM D2166/D2166M-13, while growing their specimens to a height of 6 cm and a diameter of 5 cm (i.e., 1.2:1 height:diameter ratio) [8]. To facilitate data comparison and reduce the effect emerging from corners in rectangular specimens, ASTM D2166/D2166M-13 was used [55]. Our cylindrical specimens were grown to attain a 2:1 height:diameter ratio after post-growth treatments, as specified by the standard. However, due to the large amount of contamination, the height had to be reduced to 11 cm before the application of post-growth treatments. Therefore, specimens were tested with a height of 11 cm for the uncompacted specimens and 5.5 cm for the compacted specimens. Since the diameter could not be reduced easily, the diameters remained the same (i.e., 7 cm), resulting in a 1.57:1 height:diameter ratio. 

The uniaxial compressive tests were performed at room temperature at the University of Akron’s Olson mechanical testing facility. The testing room had a temperature of 20.06 ± 0.39 °C and a relative humidity of 62.6 ± 1.36%. Due to the high humidity, the specimens were stored in the Biodesign lab, which had a temperature of 25.53 ± 0.20 °C and a relative humidity of 46.14 ± 0.64%. Previous research projects also performed mechanical tests on myceliated materials under ambient conditions: 23 °C [7], and 25°C for a 50% relative humidity [9]. A 5567 Instron with a loading cell of 10 kN was used for the compressive tests. Before starting the test, the loading cell was lowered onto the specimens until a load of 1 N was reached. ASTM 2166-13 requires a loading rate that will produce a strain of 0.5 to 2%/min. Yang et al., 2017 used a 2%/min loading rate and our team decided to follow the same rate [8]. The standard also specifies that the specimen should fail within 15 min, but our specimens did not fail. Elsacker et al., 2019 stopped their compressive tests when the strain produced a height deformation between 70 to 80% strain [9]. In this regard, our tests were stopped when the specimen experienced 75% deformation (i.e., strain of 0.75) or the maximum load of 10 kN was reached. The specimens’ dimensions (i.e., height, diameter, and weight) were measured according to the ASTM C303-10 to determine specimens’ density before the first test [58]. 

Since the mycelium-based specimens did not fail under the compressive load, the team decided to reload the specimens grown from *Pleurotus ostreatus* to study the hysteresis and dilatation of mycelium-based materials. Therefore, the specimens were first loaded up to a 75% height deformation (i.e., strain of 0.75) or a load of 10 kN. Once one of these limits was reached, the load was released. Specimens were left to dilate for 10 min. After 9 min of being released, the specimens’ height and diameter were measured. After 10 min of being released, the specimen was loaded a second time with an updated loading rate. The same formula was used to calculate the rate of testing (i.e., 2%/min). The height of the specimen changed between the start of each test. Thus, the rate was recalculated with the updated height for the second test. The height of all specimens was taken before and after both tests, 1 h after the end of the second test, and after 1 week of testing to study the dilatation over time. The width of the specimens was also recorded but was not useable due to the pieces falling off from the sides. The weight of the specimens was not recorded after the beginning of the test as specimens broke into multiple pieces every time they were handled. 

Different methods can be used to measure Young’s modulus. The slope of the linear portion of the stress–strain curve is commonly used to calculate Young’s modulus. Our materials did not fail, and the stress–strain curves did not possess linear portions. Elsacker et al., 2019, seemed to have used the entire stress–strain curve to calculate Youngs’ modulus [9]. Since our specimens did not fail, the resistance to the load started an exponential trend towards the middle of the test (i.e., densification). Therefore, extracting Young’s modulus from the entire deformation of the specimen seems to be incorrect. Two studies obtained Young’s moduli of myceliated specimens at 20% height deformation (i.e., strain of 0.20) [10,11], so this technique was chosen. Furthermore, the top and bottom surfaces of the myceliated specimens were not entirely flat, but highly textured. Therefore, the data collected at the beginning of the tests (up to around a strain of 0.05) only showed the compressive resistance of parts of the specimens. Calculating Young’s modulus at a strain of 0.2 (i.e., 20% height deformation) allowed the removal of all this variability in the data. To facilitate comparison between studies, Young’s modulus was still calculated with three different techniques used in the literature. First, it was calculated from the slope of the stress–strain curve between strain values of 0.19 and 0.20. This technique shows Young’s modulus at a strain of 0.20. Second, it was calculated from the slope from the start of the test up to a strain of 0.20. Third, it was calculated from the stress–strain slope of the entire specimen deformation. Additional information about the calculation of Young’s modulus is presented in Appendix H.

#### 2.6.2. Three-Point Bending Testing

The bending testing followed the procedure from ASTM D1037-12: Standard Test Methods for Evaluating Properties of Wood-base Fiber and Particle Panel Materials, Section 9. Static Bending [54]. Since each mold contained six specimens grown as one entity, each specimen was cut to the desired size (i.e., 76 mm) prior to testing. 

The bending tests were performed with the same Instron that was used for compression tests. A 5567 Instron with a loading cell of 100 N was used for the bending tests. A specific testing apparatus was built according to the ASTM standard. Rounded supports had a span of 266 mm, which is equal to 24 times the average nominal thickness of the panels. Before starting the test, the upper point was lowered onto the specimens until a load of 0.01 N was reached. ASTM D1037-12 requires a uniform loading rate to achieve an outer fiber strain rate of 0.005 mm/mm/min. Since each specimen had a slightly different thickness, each specimen was tested with a different loading rate. As a reference, the average loading rate of all specimens tested in bending for the first batch was 5.043 mm/min. Tests were stopped once the specimen failed, or the load applied reached less than 20% of the maximal load achieved during the test. 

**Figure A1:**
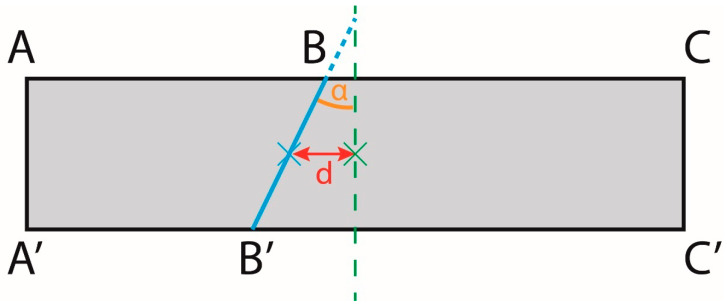
This abstract diagram shows the measurements used to compare the fracture observed in each specimen under bending to the ideal scenario. A bending specimen is displayed from the top view. The failure of the specimen (i.e., fracture) is shown in blue, whereas the ideal failure scenario is shown as a green dashed line. The two broken halves of the specimens are measured (A, B, C, A′, B′, and C′) to find the location of the specimen’s failure. The fracture offset from center (d) represents the distance between the middle of the ideal failure scenario (green point) and the middle of the actual failure location (blue point). The fracture angle is measured by comparing the axes of the ideal and actual failures (α).

The specimens’ dimensions were measured according to the ASTM C303-10 to determine specimens’ density before testing [58]. Some specimens broke asymmetrically (i.e., they did not fracture at the location of the upper point pushing down onto the specimen or the middle of the specimen) and/or the fracture was not parallel to the apparatus supports. After the test, the fracture location and angle were measured to study asymmetric failure (Figure A1). Additional information about the bending test data analysis is provided in Appendix H.

## 3. Results

### 3.1. Quantifying Particle Sizes

**Figure 2:**
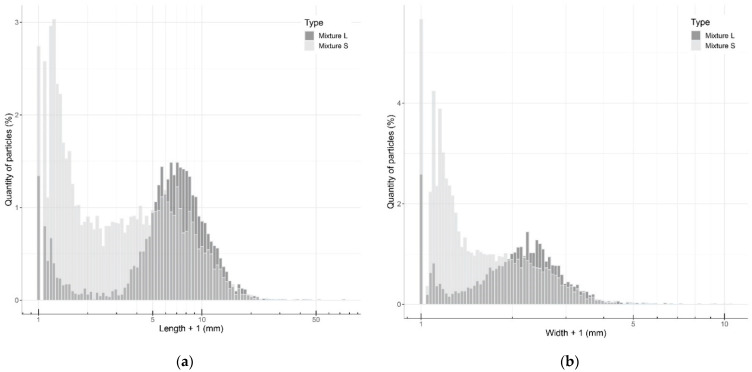
This figure shows the difference between the distribution particles’ dimensions in both mixtures (L for large and S for small). The *x*-axis is displayed on a log-scale. Due to the presence of 0s in the data (emerging from micro-particles), which equal -∞ on a log-scale, the data was offset by adding 1 to every value. Particles shown at 1 mm represent any micro-particles detected measuring less than 0.01 mm; (**a**) shows the distribution of particles’ length + 1 in both mixtures; (**b**) shows the distribution of particles’ width + 1 in both mixtures.

A total of 4004 particles were measured for the large mixture and 7014 for the small mixture. In both mixtures, micro-particles (i.e., size less than 0.01 mm) were detected. Due to reorientation of the particles during the sieving process, a few particles longer than 20 mm were still present in both mixtures. However, there was a clear difference in the lengths and widths of the set of particles from both mixtures (Figure 2). For the large mixture, the mean length of particles was 5.89 ± 4.08 mm, with a length ranging from less than 0.01 to 51.78 mm. The mean width was 0.63 ± 0.69 mm, with values ranging from less than 0.01 to 8.29 mm. For the small mixture, the mean length of particles was 2.99 ± 3.57 mm, with lengths ranging from less than 0.01 to 72.95 mm. The mean width was 1.13 ± 0.80 mm with values ranging from less than 0.01 to 9.50 mm.

### 3.2. Growth of Mycelium-Based Materials with Pleurotus ostreatus

#### 3.2.1. Growth Assessment 

Appendix I contains information about the evolution of mycelium and contaminants’ growth over time. All specimens grown for bending testing, and 65% of those for compression testing, exhibited some contamination by species believed to be *Trichoderma harzianum* or *Penicillium* sp. In addition, species visually resembling *Rhizopus* sp. (i.e., pin mold), and *Dactylium* sp. or *Hypomyces* sp. (i.e., cobweb mold), were also observed on some of the specimens. The contamination may have resulted from autoclaving large quantities of substrate or the mixing and placement in the molds (i.e., warehouse environment). The specimens grown in PVC pipes having a height of 34 cm had more contamination at the bottom of the specimen than the top, which may have resulted from higher humidity. Therefore, the growth of contaminants seems to correlate with high humidity. Throughout the growth and drying periods, fruiting bodies emerged from the specimens. These mushrooms had to be removed regularly as contaminants were growing on them. For the specimens grown for compression testing, the weight of contaminated material removed accounted for 59.16% of the initial weight from mixture L and 63.22% of that from mixture S. For the specimens grown for bending testing, the weight of material removed accounted for 40.99% of the initial weight from mixture L and 21.72% of that from mixture S. Therefore, one particle size did not seem to increase contamination in all cases (i.e., both compression and bending specimens). 

In addition to the contamination, quantities of 805 and 697 g of fruiting bodies were removed from the specimens grown for compression and bending testing, respectively. Therefore, the weight of fruiting bodies collected equaled approximatively 3.07 ± 3.48% and 3.20 ± 2.96% of the initial specimen weight for both compression and bending specimens, respectively. Since the mixture was composed of 67.5wt% of water, the weight of fruiting bodies accounted for 9.45 ± 10.70% and 9.85 ± 9.10% of the initial dry weight of the specimens, respectively.

#### 3.2.2. Shrinkage and Weight Reduction of Cylindrical Specimens Grown for Compression Testing

The shrinkage of the specimens was calculated for the entire manufacturing process. Detailed data about the shrinkage of the cylindrical specimens are shown in Appendix I. For these specimens, the mycelial growth process lowered the initial weight by 5% (Table A1). The specimens had to be slowly dried before applying the post-growth treatments. This step was necessary because post-growth treatments were applied in a non-controlled environment (i.e., warehouse) where specimens were exposed to various contaminating species. Furthermore, the materials were stuck inside their mold at the end of the growth process and could not be extracted without damaging them. Therefore, the cylindrical specimens were kept inside their mold during the drying period. This drying process reduced the weight of the specimens to 50% of their original weight. The compaction process did not significantly affect the weight of the specimens. The drying and baking treatments finally brought the weight of the materials down to around 22% of their initial weight at inoculation. 

In terms of height, the growth process did not significantly modify the specimens’ height. The drying period lowered their height by around 5%. After this drying period, the specimens were cut to a height of 11 cm. Therefore, the rest of the height evolution was considered to start with 11 cm equaling 100% at this stage. Compacted specimens were mechanically compacted to half of their height (i.e., 50% or 5.5 cm). Then, the baking and drying treatments reduced their height to 49.5% before testing. For the uncompacted specimens, the drying/baking process only reduced their height by around 0.3%. Therefore, there was a mean height shrinkage of 5.4% for the cylindrical specimens.

Shrinkage also happened horizontally (i.e., to the diameter of the cylindrical specimens). No shrinkage was observed during the growth period (Table A1). The drying period prior to post-growth treatments shrank the 17 cm high specimens (i.e., uncompacted) by an average of 3.24% An additional 0.28% diameter shrinkage was observed during the drying/baking process. The compacted specimens were compacted inside their PVC molds used during the growth period. Compaction treatment increased the specimens’ width to their original width by pressing the material against the inner surface of the molds. The specimens were removed from the molds for the baking/drying process. During this process, their diameter increased by around 5.31%. Therefore, only uncompacted specimens should be used to estimate the shrinkage observed in cylindrical specimens. The total shrinkage from inoculation to the beginning of mechanical testing would then average 3.52%.

#### 3.2.3. Shrinkage and Weight Reduction of Panel Specimens Grown for Bending Testing

Compared to the cylindrical specimen analysis, the high level of contamination made the weight study inconclusive. The uncompacted specimens’ height shrank by around 9.29% when considering all specimens (Table A2). The height of the compacted specimens was reduced by 62.68% during the entire process, including the compaction post-growth treatment. Knowing that the compaction post-growth treatment accounted for 50% of this height reduction, an average shrinkage of 12.68% was observed. In terms of horizontal shrinkage, the width of the uncompacted specimens was reduced by around 4.93%. The length shrinkage, however, could not be calculated because uncompacted specimens had to be trimmed due to contamination. Additional data about the shrinkage of the rectangular specimens are shown in Appendix I.

### 3.3. Growth of Mycelium-Based Materials with Coprinus comatus

In contrast to the first batch, mixtures L and S of the second batch contained different amounts of contamination. All specimens from mixture L were fully contaminated, and only displayed nil to low amounts of *Coprinus comatus* mycelium growth. In addition to the same species contaminating the first batch, materials grown from mixture L of the second batch also contained slime mold. All these specimens were documented and directly discarded to reduce the spread of contaminants to other specimens. In comparison, specimens grown from mixture S only displayed very low amounts of contamination across all specimens and were covered with mycelium. A share of 44% of the panels grown for bending, and 25% of the specimens grown for compression testing, exhibited some contamination. However, the levels of contamination were very low compared to the first batch, and no contamination was removed from the specimens. The difference in contamination levels is likely due to the pasteurization process not being successful for mixture L. The same pasteurization procedure was used for both mixtures, with the only difference being that mixture L was pasteurized before mixture S. In summary, all specimens grown from mixture L had to be thrown away due to the high levels of contamination and lack of *Coprinus comatus* mycelial growth. In contrast, all materials grown from mixture S were covered with mycelium and tested with no contamination removed.

### 3.4. Mechanical Testing

The mechanical testing served to compare the effects of three main variables on the bio-composite material’s properties: fungal species (*Pleurotus ostreatus* and *Coprinus comatus*), substrate particle sizes (with or without micro-particles), and post-growth treatment (dried, baked, compacted then dried, and compacted then baked). The acronyms given to each sample are shown in Table 1.

#### 3.4.1. Compression Testing

Of the 40 specimens grown with *Pleurotus ostreatus* (first batch) for compression tests, 26 were useable for testing due to the removal of contaminated specimens throughout all experimental groups. In this regard, the sample size of each experimental group was reduced from five to three or four depending on the group. By comparison, for the second batch, all mycelium-based materials grown from mixture S were usable (sample size of 10), but none from mixture L. Therefore, the comparison between both mixtures could not be performed for the *Coprinus comatus* species. Young’s modulus of each sample is shown in Table 2. No specimen experienced sudden failure, as they were all compressed throughout the entirety of the test (i.e., until a strain of 0.75 or a load of 10 kN was reached). The compressive Young’s moduli were calculated following three methods. As stated in Section 2.6.1, the method considering the linear slope between a strain of 0.19 to 0.21 was selected for this analysis (bold in Table 2). The compressive Young’s modulus ranged from 0.15 MPa (PO_LNCB) to 4.55 MPa (PO_SCB). A positive correlation between the density of specimens and their compressive Young’s modulus was observed for all samples, except one (PO_SNCD). There was no significant difference between the compressive Young’s modulus of the materials grown from *Pleurotus ostreatus* and *Coprinus comatus*. 

**Figure 3:**
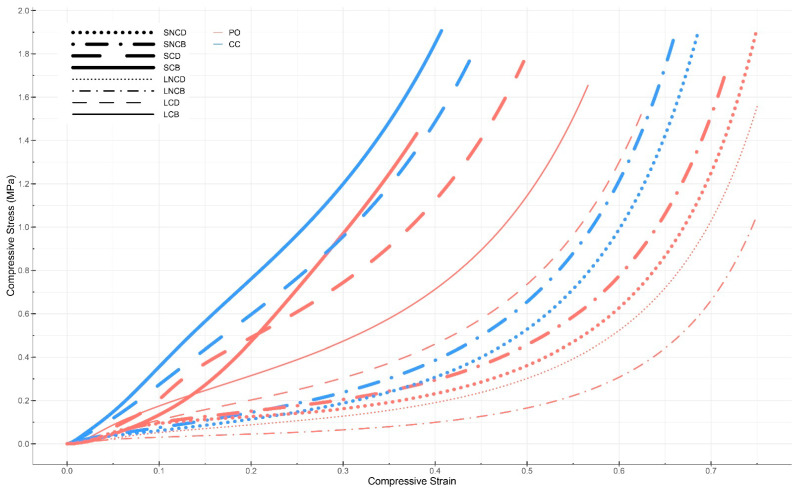
This figure shows the mean stress–strain curves for each sample of mycelium-based materials. The stress required to compress the material (i.e., increasing strain) increases linearly then exponentially. Due to the high variability in the stress–strain slope, the Young’s modulus was calculated from 3 different strains as shown in Table 2. The Young’s modulus used for comparison was calculated from 0.19 to 0.21 strain, to represent the slope of the stress/strain curve at 0.20 strain. “PO” stands for samples grown from *Pleurotus ostreatus*, and “CC” for *Coprinus comatus*. Acronyms for each sample are labeled in Table 1.

**Figure 4:**
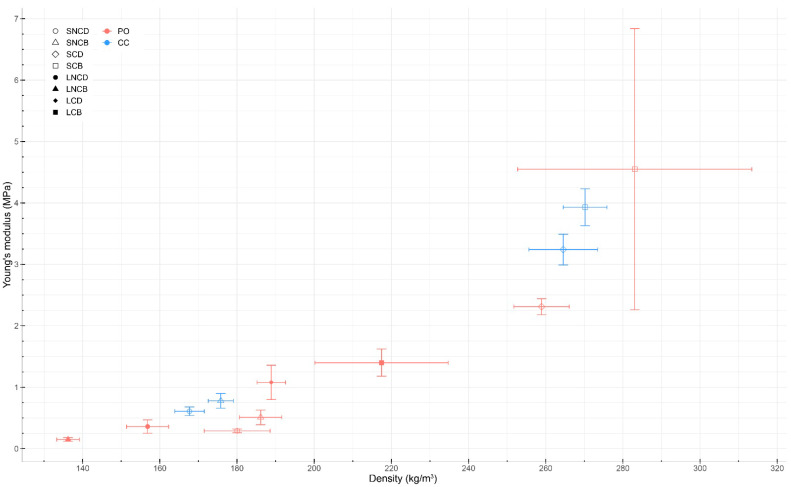
This figure shows the mean Young’s moduli of the samples in relation to their mean density before compression testing. Young’s moduli represented in this figure were calculated from 0.19 to 0.21 strain as it is closest to the traditional calculation of the Young’s modulus. “PO” stands for samples grown from *Pleurotus ostreatus*, and “CC” for *Coprinus comatus*. Acronyms for each sample are labeled in Table 1.

In our first batch grown from *Pleurotus ostreatus*, the compressive Young’s moduli of the samples ranged from 0.15 to 4.55 MPa. Specimens (PO_LNCB) made from mixture L (substrate without fine particles) that were non-compacted and baked resulted in the lowest average Young’s modulus at 0.15 MPa for a density of 136.22 kg/m^3^. Specimens (PO_SCB) made from mixture S (substrate with fine particles) that were compacted and baked resulted in the highest average Young’s modulus at 4.55 MPa. These specimens had a mean density of 283.07 kg/m^3^. Overall, the average Young’s moduli of the samples made from mixture S ranged from 0.29 to 4.55 MPa. The Young’s moduli and stress–strain curves of the various samples grown from *Pleurotus ostreatus* show multiple trends (Figure 3 and Figure 4): first, there was a positive correlation between the density of the samples and their compressive Young’s moduli. Only one sample (PO_SNCD) of eight did not follow this correlation. Second, pre-compacting the myceliated specimens before the compression testing increased their Young’s moduli as the four highest moduli were extracted from pre-compacted samples. Third, for the same post-growth treatments, specimens made out of mixture S (with fine particles) had higher Young’s moduli than those from mixture L (without fine particles). Fourth, the baked materials had a higher Young’s moduli than dried ones in three cases of four. The exception was the Young’s modulus of LNCD being higher than that of LNCB. Finally, the mean relative humidity of the samples before testing was 8.82 ± 1.23%. The low variation in the relative humidity of the samples, especially due to the lack of correlation with the Young’s moduli, did not likely affect the results of these compressive tests. The difference in relative humidity between samples is likely related to the post-growth treatment used. Baking resulted in a lower relative humidity than drying for 3 samples out of 4. However, the standard deviations show variations within each sample. Therefore, differences in relative humidity could also have emerged from variations in material heterogeneity (i.e., amount of mycelium growth, and distribution and orientation of particles). Since the weight of fruiting bodies harvested on each specimen was collected, the impact of fruiting body formation on mechanical performance of the composite material could be addressed. However, these results should be considered with care as they emerged from a study that was not designed for addressing such impact. Larger amounts of fruiting bodies harvested led to slightly lower Young’s modulus in five of eight samples.

In the second batch grown from *Coprinus comatus*, the Young’s moduli of specimens made from mixture S (substrate with fine particles) ranged from 0.58 to 3.69 MPa. Specimens (CC_SCB) that were compacted and baked had the highest average Young’s modulus at 3.69 MPa with a mean density of 270.21 kg/m^3^. Similar trends were observed in compression tests of materials grown from both species (Figure 3 and Figure 4). There was a positive correlation between the samples’ densities and their compressive Young’s moduli. Compacted samples had higher Young’s moduli than non-compacted ones. In both cases, baked materials showed a higher Young’s modulus than dried ones. For non-compacted specimens, the difference between baking and drying was small. For compacted specimens, similar to the previous batch, baking resulted in higher Young’s moduli than drying. However, the humidity of the baked samples was slightly lower than that of the dried samples. The mean relative humidity of the samples before testing was 5.86 ± 0.97%. The high sample size (i.e., 10) increases the validity of these results compared to those of the previous batch.

**Figure 5:**
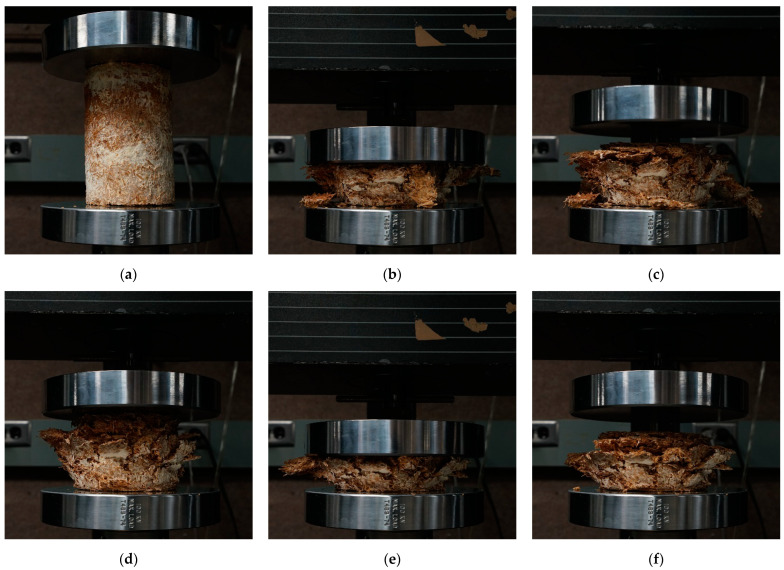
This figure shows the deformation of a specimen throughout the compression test: (**a**) beginning of the test; (**b**) end of the first test where a compressive strain of 0.75 was reached; (**c**) 5 s after removing the load from the first test; (**d**) 10 min after removing the load from the first test which equals the beginning of the second test; (**e**) end of the second test where a compressive strain of 0.62 and a load of 10 kN were reached; (**f**) 5 s after removing the load from the second test.

The study of materials’ hysteresis showed that material height after test 2 was slightly lower than that after test 1 (Table 3 and Figure 5). Therefore, the material was still compressible after the first test (i.e., strain of 0.75 or 10 kN reached). In terms of dilatation, specimens did not dilate back to their initial height prior to testing (Table 3). During the first test, most uncompacted specimens reached the 0.75 strain limit first, while most of the pre-compacted ones reached the 10 kN limit first. For the second test, the maximal load of 10 kN was reached before 0.75 strain for all specimens. After the second test, most of the dilatation occurred within the first hour. Some dilatation was already observed 5 s after load removal (Figure 5). However, dilatation still occurred beyond one hour after compressive testing. A week after testing, pre-compacted specimens dilated back to a height closer to their initial height, in comparison to uncompacted specimens. In conclusion, both elastic and plastic deformations occurred in mycelium-based materials as all specimens experienced some dilatation after testing.

#### 3.4.2. Bending Testing

**Figure 6:**
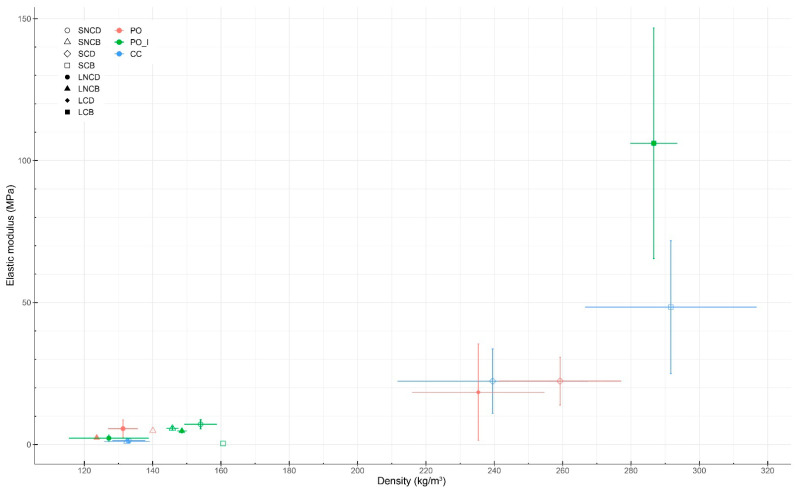
This figure shows the modulus of elasticity of samples in relation to their density prior to testing. “PO” stands for samples grown from *Pleurotus ostreatus* and the added “_I” means that corresponding samples were grown as extras under different growth environments, mold sizes, and timelines. “CC” refers to *Coprinus comatus* samples. Acronyms for each sample are labeled in Table 1.

For bending tests, 17 of 48 specimens grown with *Pleurotus ostreatus* (first batch) were useable for testing. Due to high levels of contamination throughout growth, extra specimens, grown from leftover mixtures in different molds, had to be cut down and used for bending testing. However, these extra specimens were not grown in the same environments nor processed along the same timeline. They were marked with the letter “I” to separate them from the regular bending specimens (e.g., PO_ILNCB and PO_ISCB). Therefore, the sample size of available specimens for testing ranged from 0 to 6 when considering both original and extra specimens separately (Table 4). For materials grown with *Coprinus comatus*, 52 of 54 mycelium-based materials grown from mixture S were usable, leading to sample sizes ranging from 11 to 15. Similar to specimens tested in compression, no specimens from mixture L were usable for bending testing. The comparison between both mixtures was again only possible for the *Pleurotus ostreatus* species. The elastic moduli of all samples are shown in Table 4 and Figure 6. No significant difference was observed between the elastic moduli of samples grown from the two fungal species. Each wooden mold contained six specimens, which were cut prior to testing. The edges of myceliated panels often exhibited more mycelial growth than their inside. Therefore, specimens on the edge of the panels were tracked. Yet, no significant difference in the elastic modulus was observed between edge and central specimens across all samples. 

For specimens from the first batch (grown with *Pleurotus ostreatus*) tested in bending, contradicting results were observed. However, specimens grown as extras were used due to high levels of contamination and resulting low sample size. Since they were grown in molds of a different size and processed on a delayed timeline, results should be analyzed with care. Therefore, data comparison between original specimens is more reliable than comparison between specimens grown as extras. Comparisons between original and extra specimens are only shown when the variable addressed cannot be analyzed between original or extra specimens. For instance, specimens (PO_ILCB) made from mixture L (substrate without fine particles), which were compacted and baked, resulted in the highest average elastic modulus at 106.08 ± 40.65 MPa for a density of 286.67 kg/m^3^. In comparison, the rest of the samples ranged from 0.40 MPa (PO_ISCB) to 22.39 MPa (PO_SCD). One specimen (PO_ISCB) made from mixture S (substrate with fine particles), which was compacted and baked, resulted in the lowest average elastic modulus at 0.40 MPa for a density of 160.60 kg/m^3^. This result is in contradiction with the trends observed across other samples and may be due to the different process used since this panel was grown as an extra from leftover mixture. In terms of variables, mixture S resulted in higher elastic moduli than mixture L in two samples out of two (PO_NCB and PO_CD) for the original specimens; and three samples out of four (PO_INCB, PO_INCD, PO_ICD and PO_ICB) for panels grown as extra. Drying resulted in higher moduli of elasticity than baking in one sample out of one (PO_LNC) for original specimens; one sample out of two (PO_ILNC and PO_ISNC) for panels grown as extra; and one sample out of two (PO_LC/PO_ILC and PO_SC/PO_ISC) when comparing original and extra specimens. Compacting led to higher moduli of elasticity in one sample out of one (PO_LD) for original specimens; one sample out of two (PO_ISB and PO_ILB) for panels grown as extra; and one sample out of one (PO_SD/PO_ISD) when comparing original and extra specimens. Such results should be interpreted with care since sample sizes ranged from one to six specimens. In conclusion, variables that led to higher elastic moduli were mixture S and compaction. The difference between the effects of drying or baking was less pronounced. For each sample tested under bending, specimens (i.e., that received the same treatments) were grown in the same mold. The quantity of fruiting bodies harvested was recorded for each mold. Therefore, no data analysis on fruiting bodies’ effect on bending properties could be performed.

In the second batch, elastic moduli of mycelium-based samples made of *Coprinus comatus* grown on mixture S (with fine particles) ranged from 1.16 to 48.39 MPa. Specimens (CC_SNCB) that were not compacted and baked resulted in the lowest average elastic modulus at 1.16 ± 0.54 MPa for an average density of 132.49 kg/m^3^. Specimens (CC_SCB) that were compacted and baked resulted in the highest average elastic modulus at 48.39 ± 23.37 MPa for an average density of 291.66 kg/m^3^. In both cases, compacted specimens had substantially higher moduli of elasticity than non-compacted equivalents. Baking resulted in a higher modulus of elasticity than drying only for compacted specimens. This increase in modulus due to baking can partially be explained by an increase in density for these baked specimens. For non-compacted specimens, the process of baking or drying did not have a significant effect on their elastic modulus. 

**Figure 7:**
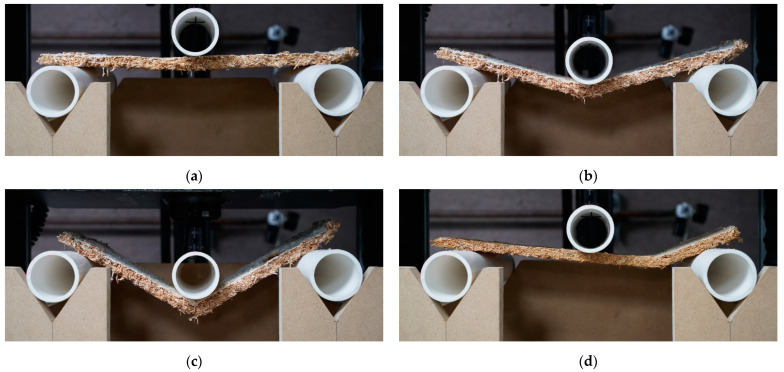
This figure shows the deformation of a PO_LNCB specimen throughout the bending test: (**a**) beginning of the test; (**b**) after 6 min and 25 s; (**c**) after 13 min (just before the end of the test). (**d**) shows the asymmetric deformation of a different specimen from the PO_LCD sample after 3 min.

**Figure 8:**
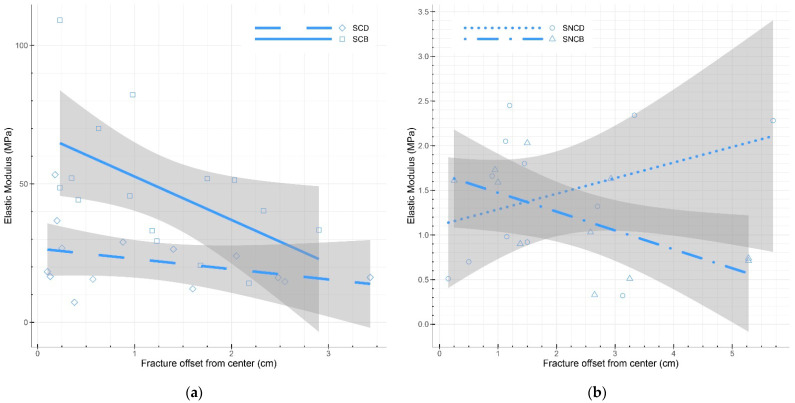
This figure shows elastic moduli calculated from the ASTM D1037 formula in relation to the distance between the fracture and the center of the specimen, grown from *Coprinus comatus*: (**a**) compacted samples; (**b**) uncompacted samples. For the compacted and baked non-compacted samples, elastic modulus decreases as fracture happens further away from the ideal scenario. Acronyms for each sample are labeled in Table 1.

For all specimens, the location of failure (i.e., fracture) under the bending load was recorded (Table 4 and Figure A1). In a three-point bending test, failure should occur in the middle of the specimen. This fracture should also be parallel to the supports bending the specimen, so the angle between the axis of the fracture and the axis of the supports (i.e., its width as the supports were parallel to the width of the specimens) was calculated (Figure A1). Fracture location varied throughout all samples grown from both species (Figure 7), with no consistent trend observed (Table 4). In specimens grown from *Pleurotus ostreatus*, fracture angle heavily varied in comparison to the ideal failure scenario, ranging from 2.19° (PO_SNCB) to 13.35° (PO_LNCD) (Table 4). By comparison, specimens grown from *Coprinus comatus* failed at an angle close to the ideal scenario as fracture angle ranged from 0.51° (CC_SNCD) to 0.67° (CC_SCB). Within compacted specimens grown from *Coprinus comatus*, there was a decrease in modulus of elasticity as fracture happened further away from the specimen’s center, especially in baked specimens (CC_SCB) (Figure 8). Therefore, location and angle of failure can be indicators of decreasing resistance under bending. 

## 4. Discussion

### 4.1. Mycelial Growth

#### 4.1.1. Reducing Contamination and Promote Mycelial Growth

The mycelium-based materials recipe and processes impact the growth of contamination and mycelium, which impacts the performance of the materials. For the two batches grown in this study, the substrate was either sterilized (batch 1) or pasteurized (batch 2). Pasteurization is known to kill pests and competitors while minimizing the loss of beneficial micro-organisms [59]. In this regard, pasteurization resulted in lower contamination levels during mycelium growth over the substrate than sterilization [5]. In our study, medium levels of contamination were observed throughout all specimens during mycelium growth over sterilized substrate (i.e., both mixtures of batch 1). By comparison, specimens grown with pasteurized substrate (i.e., batch 2) were either fully contaminated (i.e., those grown with mixture L) or only displayed nil to very low amounts of contamination (i.e., those grown with mixture S containing fine particles). Since no significant difference in contamination was observed between specimens grown with both mixtures in batch 1, and mixtures with fine particles are harder to sterilize or pasteurize than those without, it seems unlikely that the mixture was the cause of the contamination levels. The variation in contamination levels between mixtures of batch 2 was most probably due to the success of the pasteurization process. Even if both sterilization/pasteurization processes were conducted on two different fungal species, pasteurization seems more effective as it can inhibit all contamination if successful [5]. The absence of contamination increases the process’ potential for replication. In conclusion, future research should prioritize pasteurization of the substrate.

Furthermore, the growth of contaminants competes with the growth of the inoculated fungal mycelium species. Existing research projects have shown variations in growth time ranging from 6 days up to months [7,13,34,35]. In our case, access to the growth lab was limited due to COVID-19 regulations. Twenty-one days after inoculation, a thin mycelium mat was observed over most of the specimens’ visible surfaces. The growth time was set to 46 days for both batches to ensure complete growth over all specimens. The addition of more-nutritious substrates or higher concentrations of mycelium spawn enhances mycelial growth and reduces contamination, resulting in higher mechanical properties [13]. However, this addition (not used in this project) decreases the sustainability of these materials as the substrate is composed of products other than waste or byproducts. 

#### 4.1.2. Food Production

During the growth and drying phases, fruiting bodies were collected on the mycelium-based materials grown with *Pleurotus ostreatus*, but not on those grown with *Coprinus comatus*. Yet, materials were grown in the same dark environment with the same temperature and relative humidity. However, they were not grown during the same season (i.e., March–May for *Pleurotus ostreatus* and June–August for *Coprinus comatus*). The formation of *Pleurotus ostreatus* fruiting bodies is most abundant in spring in temperatures ranging from 4 to 24 °C [51], which is the season in which our materials were grown. *Coprinus comatus* fruiting bodies are most prolific under spring and fall temperatures ranging from 4 to 16 °C [51], whereas our materials were grown in the summer. Therefore, the variation in mushroom yield may result from the difference in the fungal species and/or growth season.

The weight of *Pleurotus ostreatus* fruiting bodies harvested from compression and bending specimens, respectively, equaled 9.45 ± 10.70% and 9.85 ± 9.10% of the initial specimens’ weight. Therefore, the biological efficiency (i.e., the effectiveness of the process for mushroom production) equaled 9.45% for the compression specimens and 9.85% for the bending specimens. A wide variation of yield was observed among our specimens. The biological efficiency ranged from 0.00 to 35.94% for the compression specimens and from 0.00 to 20.51% for the bending specimens. As a reference, the biological efficiency of various substrates grown with the same fungal species, *Pleurotus ostreatus*, ranged from none for elephant grass to 61.04% for composted sawdust [60]. However, our process mainly focused on producing mycelium-based materials and was not optimized for mushroom yield. Materials were grown inside a PVC tube or a flat rectangular mold enclosed with housewrap in a dark environment. Fruiting bodies were able to grow through the housewrap material that sealed the PVC tubes by enlarging the small holes already present in this material. Therefore, they could be harvested without removing the housewrap and exposing the mycelium-based material to potential contaminants. Mushrooms were not harvested in multiple flushes and their growth was stopped by drying the materials. Therefore, the biological efficiency is presented as a reference and only shows the potential of combined material and food production. 

#### 4.1.3. Material Shrinkage

Cylindrical specimens grown for compression testing shrank, on average, by 5.4% of their original height. These specimens measured 17 or 34 cm in height and were grown and dried inside PVC tubes. The specimens grown were taller than those produced in previous research projects and were dried inside their mold (i.e., PVC pipes). This set-up may have resulted in friction between the specimen and the internal walls of their mold, thus limiting shrinkage. In terms of horizontal shrinkage (i.e., in diameter), the shrinkage of uncompacted specimens from inoculation to the beginning of the mechanical testing was approximatively 3.52%.

For the rectangular specimens grown for bending testing, compacted materials shrank by an average of 12.68% in addition to the 50% compaction. Uncompacted specimens shrank by 9.29%. The specimens’ width was reduced by around 4.93% for the uncompacted specimens. The lower shrinkage in width compared to height likely resulted from the friction caused by the bottom of the molds.

### 4.2. Compression Testing

In our analysis, the fungal species did not have a distinct effect on the compressive Young’s modulus of the mycelium-based materials. In the literature, fungal species have been shown to affect the mechanical properties of mycelium-based materials. For instance, *Ganoderma* spp. generally have a higher strength than *Pleurotus* spp. [12], but Haneef et al. found contradictory results [7]. The effect of fungal species seems to depend on the hyphal types (i.e., monomitic, dimitic, and trimitic), which can be generative, binding, and/or skeletal [30,31]. 

Compressive Young’s modulus was positively correlated with sample density. Density is known to have a significant effect on the stress–strain curve response of mycelium materials [48]. The correlation between porosity/density and compressive modulus was previously discussed in [13]. The density of the final mycelium-based materials can be controlled in two main steps of the process: during substrate processing and application of post-growth treatments. For example, sawdust is known to be denser than straw. However, as mycelium growth is based on oxygen access, it is usually reduced in the center of dense substrates [5,13]. By comparison, compaction after mycelial growth will partly break the mycelium network. Therefore, a trade-off between both options could be further studied. When comparing materials grown from *Pleurotus ostreatus*, materials made from mixture S (substrate with fine particles) showed an increase in density by 30% and resulting increase in compressive Young’s modulus by 156% compared to those made from mixture L (without fine particles). In comparison, Rigobello and Ayres found that materials made from substrate particles having sizes ranging from 0.5 to 12.0 mm had a slightly higher density but lower Young’s modulus than those made from particles having sizes ranging from 4.0 to 12.0 mm [42]. In their case, the presence of smaller particles reduced Young’s modulus. In terms of post-growth treatments, material compaction increased density by 48% when considering all specimens but increased compressive Young’s modulus by 511%. For structural applications, the material should be compacted after the growth of mycelium to increase its compressive modulus while avoiding mycelium growth inhibition emerging from a lack of oxygen access. When averaging all samples, baking increased material density by 4% but increased compressive Young’s modulus by 43%. Baking (at 100 °C) is supposed to kill the fungus that may damage the mycelial network throughout the specimen. By comparison, drying (at 40 °C) is only supposed to put the fungus in a dormant stage. In this regard, the choice of drying or baking the materials depends on the desired application. For instance, keeping the fungus alive can potentially induce self-healing properties, but may not be desired in environments where humans may potentially be exposed to large quantities of fungal spores. The slight humidity difference between samples may be another factor influencing the variation in Young’s modulus, since the strength of a material is roughly inversely proportional to its moisture content [45]. However, no correlation was observed between those values.

The substrate preparation, growth conditions, and post-growth processes used in this research resulted in similar or slightly higher compressive Young’s moduli than those recorded in previous research projects, as described in Section 1. Introduction [8,9,11,31,45,48]. For instance, materials grown from *Pleurotus ostreatus* on hemp mat reached a compressive strength of 0.19 MPa [31]. In comparison, our mycelium-based samples made of *Pleurotus ostreatus* grown on chipped and sieved straw resulted in compressive Young’s moduli ranging from 0.15 to 4.55 MPa. To the authors’ knowledge, no studies have tested the mechanical properties of materials grown with *Coprinus comatus*. Current mycelium research explores the use of mycelium-based materials to replace traditional foam materials. In this case, the goal is to obtain high mechanical strength with low density. However, difficulties in manufacturing lead to inconsistencies in mechanical behavior of composite materials. For instance, materials made from the same substrate still show large standard deviations due to varying particle distribution and orientation [42].

Studying the hysteresis and dilatation of mycelium-based materials is important to predict their final dimensions and behavior. Since the height of all specimens increased after the compressive tests, materials experienced some elastic deformation. In addition, since no specimen dilated back to their initial height, plastic deformation also occurred in all materials tested. Drying the grown materials seemed to increase dilatation after compression of materials made from the large mixture. For specimens grown with the small mixture (i.e., containing fine particles), baking resulted in increased dilatation after compression testing. As expected, specimens that experienced higher strain (i.e., uncompacted specimens) were less likely to dilate back to a height closer to their initial height. Depending on the application, various substrate mixtures and post-growth treatments can be employed to target desired material behavior under compression, including maximum load, elasticity, hysteresis, and dilatation.

### 4.3. Bending Testing

Similar to compression testing, the elastic modulus of the mycelium-based materials was not thoroughly affected by the difference in fungal species, but the modulus was strongly correlated with material density. The results from the *Pleurotus ostreatus* materials were less consistent than those grown from *Coprinus comatus*. The main difference in behavior between the two fungal species was observed in the failure angle. Even if the specimens of each sample were produced with the same process, the variation in elastic moduli remained high, especially for materials grown from *Pleurotus ostreatus*. The variations in elastic moduli and failure angle among samples may partially be explained by the heterogeneous properties of the material due to irregular substrate particle distribution and orientation, along with heterogeneous mycelium growth. Furthermore, results obtained from the bending materials grown with *Pleurotus ostreatus* should be interpreted with care due to the small sample size and testing of materials initially grown as extra. 

On average, the presence of fine particles in the substrate mixture resulted in a higher density, yielding higher elastic moduli. The difference between drying and baking did not have a significant effect on the material’s elastic modulus, whereas compaction substantially increased the elastic modulus. For instance, the compaction of materials grown from *Coprinus comatus* resulted in a density increase of 100% and an increase in the elastic modulus of 2611%. In comparison to Appels et al., 2019, our compacted then baked specimens achieved flexural moduli in the same range as those of heat-pressed specimens having higher densities [32]. Therefore, the substrate preparation and post-growth treatments can be used to tune the behavior of the materials under bending for the application of interest. 

### 4.4. Emerging Synergies between Bioremediation, and the Production of Food, Materials, and Medicine

This project is a first step towards a model in which fungi, especially their mycelium, can serve to decontaminate substrates, while producing fruiting bodies, medicinal drugs, fertilizers, and materials (e.g., for buildings or packaging). It stems from a circular model described by Paul Stamets, in which fungi are used for food, medicine, bioremediation, and fertilizer [51]. The updated model can be implemented in the two following examples.

The first example aims at reducing the environmental dispersal of chemicals that negatively impact the environment. The run-off of nitrates and phosphates, coming from agricultural fertilizers, is the main cause of harmful algae blooms in the Great Lakes [61,62]. The bloom of harmful algae and cyanobacteria lowers water oxygen levels, kills marine life, and negatively affects human health and agriculture [63]. Furthermore, the dispersal in the environment of these limited resources contributes to their increasing scarcity. As a response, hyper-accumulators of nitrates and phosphates, in the form of prairie grasses, could be planted along the edge of over-fertilized agricultural fields to limit environmental dispersal of these chemicals. The prairie grasses could then be harvested according to the growing seasons. They would then serve as a substrate for fungal growth to filter and uptake the substances, thus limiting their return in the environment. A variety of fungal species could be grown on this substrate to provide various functions, such as harvesting scarce substances in the fruiting bodies, producing mycelium-based materials, supplying food through edible fruiting bodies, and manufacturing of medicinal drugs. Chemical testing would be conducted throughout the process to track the substances and ensure safe levels depending on the targeted application. At the end of their life cycle, mycelium-based materials are biodegradable and can be reused as compost for fertilizers or as substrate for new generations of mycelium growth. Furthermore, a wider variety of organic substrates could be used to increase the impact of this upcycling process. For instance, algae can be harvested using algal turf scrubbers to produce living materials and various substances [4,64,65,66,67,68].

Similarly, organic waste from building and demolition sites (e.g., wood) can serve as substrate for mycelium growth to produce food and materials while decontaminating it. A large quantity of waste placed in landfills comes from construction and demolition sites, and its toxicity can contaminate the environment, including ground water [2,3]. Various studies have shown that children from various countries, such as USA, Nigeria, and France, are being poisoned with lead from buildings, especially through paint degrading into dust particles [69,70,71,72,73,74]. As a response, various fungal species can serve to substantially reduce lead concentration in aqueous substrates [75]. Fungi secrete enzymes known to break down aromatic compounds, including Polycyclic Aromatic Hydrocarbons (PAHs), present in waste from construction materials [76]. *Coprinus comatus*, a bio-accumulator of heavy metals, is a species recommended for decontamination of substrates with nitrates and phosphorus-bound toxins [51]. Research shows that using biochar and mycoremediation can lock away contaminants, such as lead, from the water cycle and bioavailability [75,77,78]. Chemical analysis should be performed based on the substrate and fungal species used to quantify the accumulation and breakdown of the various chemicals contaminating the substrate. Such analysis, called Toxicity Characteristic Leaching Procedures (TLCPs), would evaluate the effect of mycelium growth on bioremediation while ensuring the edibility of the fruiting bodies.

## 5. Conclusions

The diversity of fungal species and their functions has the capability to serve multiple functions in our industrialized society, from decontamination to production of food, materials, and medicine. This study shows the potential of two fungal species commonly used in bioremediation practices to produce building materials. These mycelium-based materials showed Young’s and elastic moduli comparable to or slightly higher than those from other mycelium-based materials, depending on the variables studied. The effect of the selected variables (e.g., fungal species, substrate particle size, and post-growth treatments) tested in this study enhances knowledge about which parameters serve to tune mechanical properties of the final material. Density of composite materials was positively correlated with mechanical performance. The presence of micro-particles and compaction of grown material (as a post-growth treatment) increased density and the resulting Young’s modulus, in addition to the elastic modulus. Differences between baking or drying grown material were less pronounced, with baking leading to slightly higher compressive moduli in most cases. The standard deviations observed within samples exemplify the need to better understand variables affecting material properties and to develop more accurate manufacturing procedures. Heterogeneity of produced materials was particularly apparent when analyzing the fracture location’s eccentricity of materials under bending. The study of shrinkage during growth and drying periods provides valuable insight into predicting the materials’ final dimensions. Both fungal species led to similar mechanical behavior. In addition to *Pleurotus ostreatus*, *Coprinus comatus*, a species known for bioremediation and food production, can also serve for material production and, potentially, a combination of all three. Integration of material production is suggested as an enhancement of the circular model described by Paul Stamets [51]. Further studies still need to be conducted to validate the combined use of these species’ mycelium as a binder for building materials while decontaminating substrate and producing edible fruiting bodies.

## Figures and Tables

**Table 1:** This table shows the names of the various samples produced in this study. The fungal species was added in front of these names: PO_ for *Pleurotus ostreatus* (e.g., PO_SNCD), and CC_ for *Coprinus comatus* (e.g., CC_SNCD).

Sample	Substrate Mixture	Compacted or Not	Dried or Baked
SNCD	Small (with fine particles)	Not compacted	Dried
SNCB			Baked
SCD		Compacted	Dried
SCB			Baked
LNCD	Large (without fine particles)	Not compacted	Dried
LNCB			Baked
LCD		Compacted	Dried
LCB			Baked

**Table 2:** This table shows relative humidity before testing, density, and Young’s moduli of the samples for the three different calculation techniques. Acronyms for each sample are labeled in Table 1.

Sample	Sample Size	Relative Humidity (%)	Density (kg/m^3^)	Compressive Young’s Modulus (MPa)		
				Linear from 0.00 to 0.20 Strain	Linear from 0.19 to 0.21 Strain	Linear for Entire Test
PO_SNCD	3	9.23 ± 0.79	180.04 ± 8.54	0.63 ± 0.05	**0.29 ± 0.03**	2.56 ± 0.19
PO_SNCB	3	6.50 ± 0.32	186.13 ± 5.46	0.75 ± 0.13	**0.51 ± 0.12**	2.75 ± 0.52
PO_SCD	3	10.40 ± 1.10	258.93 ± 7.17	2.45 ± 0.34	**2.31 ± 0.13**	3.63 ± 0.14
PO_SCB	3	8.80 ± 1.09	283.07 ± 30.33	2.36 ± 0.79	**4.55 ± 2.29**	4.56 ± 0.73
PO_LNCD	3	7.78 ± 0.52	156.83 ± 5.45	0.44 ± 0.11	**0.36 ± 0.11**	2.08 ± 0.55
PO_LNCB	3	8.17 ± 1.12	136.22 ± 2.94	0.23 ± 0.01	**0.15 ± 0.03**	1.41 ± 0.16
PO_LCD	3	10.67 ± 0.34	188.88 ± 3.69	1.22 ± 0.30	**1.08 ± 0.28**	2.72 ± 0.29
PO_LCB	4	9.40 ± 0.90	217.46 ± 17.26	1.58 ± 0.24	**1.40 ± 0.22**	3.24 ± 0.21
CC_SNCD	10	6.51 ± 0.23	167.69 ± 3.83	0.57 ± 0.06	**0.61 ± 0.07**	3.17 ± 0.04
CC_SNCB	10	6.05 ± 0.23	175.79 ± 3.25	0.72 ± 0.07	**0.78 ± 0.12**	3.21 ± 0.05
CC_SCD	10	6.36 ± 0.45	264.55 ± 8.90	2.98 ± 0.30	**3.24 ± 0.25**	4.42 ± 0.17
CC_SCB	10	4.52 ± 0.97	270.21 ± 5.65	3.80 ± 0.17	**3.93 ± 0.30**	4.87 ± 0.17
CC_LNCD	0	/	/	/	**/**	/
CC_LNCB	0	/	/	/	**/**	/
CC_LCD	0	/	/	/	**/**	/
CC_LCB	0	/	/	/	**/**	/

**Table 3:** This table shows height deformation or strain of samples over time throughout the mechanical testing process (i.e., before the first test, immediately after the first test, before the second test (which equaled 9 min after the end of the first test), immediately after the second test, one hour after the second test, and one week after the test). Acronyms for each sample are labeled in Table 1.

Sample	Height Deformation (%)					
	Before Test 1	After Test 1	Before Test 2	After Test 2	1 h after Test 2	1 Week after Test 2
PO_SNCD	0.00	75.00	50.44	77.87	51.50	47.88
PO_SNCB	0.00	73.81	48.20	76.61	47.35	42.32
PO_SCD	0.00	50.39	21.01	51.94	21.95	18.06
PO_SCB	0.00	47.17	20.31	48.57	20.47	16.04
PO_LNCD	0.00	75.02	/	/	45.51	38.44
PO_LNCB	0.00	75.00	48.03	83.13	55.40	52.10
PO_LCD	0.00	61.15	/	/	29.80	23.91
PO_LCB	0.00	59.25	29.68	60.71	30.52	27.68

**Table 4:** This table shows relative humidity before testing, density, and the modulus of elasticity calculated by the Instron and from the ASTM D1037 formula (bold). It also presents the maximum load sustained by all samples, the distance between the center of the specimen and the fracture location (i.e., fracture offset from center), and the angle of that fracture in comparison to the axis of the bending supports. The letter “I” in the sample name means that the corresponding specimens were grown as an extra under different growth environments, mold sizes, and timelines. Acronyms for each sample are labeled in Table 1.

Sample	Sample Size	Relative Humidity (%)	Density (kg/m^3^)	Elastic Modulus		Maximum Load (N)	Fracture Offset from Center (cm)	Fracture Angle (°)
				From Instron (MPa)	From ASTM D1037 (MPa)			
PO_SNCD	0	/	/	/	**/**	/	/	/
PO_SNCB	1	7.65	140.06	/	**4.88**	1.28	1.03	2.19
PO_SCD	6	6.03 ± 0.63	259.24 ± 17.86	31.66 ± 8.59	**22.39 ± 8.41**	3.62 ± 1.92	3.03 ± 1.86	3.60 ± 2.61
PO_SCB	0	/	/	/	**/**	/	/	/
PO_LNCD	2	5.68 ± 0.23	131.33 ± 4.36	6.37 ± 2.64	**5.64 ± 3.07**	0.64 ± 0.25	1.24 ± 0.34	13.35 ± 2.09
PO_LNCB	2	5.73 ± 0.13	123.63 ± 0.24	3.11 ± 0.29	**2.46 ± 0.23**	0.32 ± 0.08	0.81 ± 0.06	3.00 ± 2.24
PO_LCD	6	5.93 ± 0.56	235.32 ± 19.29	20.12 ± 17.44	**18.46 ± 16.95**	1.84 ± 1.48	4.29 ± 2.18	4.46 ± 3.23
PO_LCB	0	/	/	/	**/**	/	/	/
PO_ISNCD	5	5.73 ± 0.48	154.04 ± 4.73	8.91 ± 2.13	**7.17 ± 1.60**	0.90 ± 0.33	1.18 ± 0.58	4.27 ± 5.21
PO_ISNCB	3	5.65 ± 0.24	145.78 ± 1.77	6.08 ± 0.39	**5.74 ± 0.58**	0.50 ± 0.12	2.86 ± 1.51	6.84 ± 5.59
PO_ISCD	0	/	/	/	**/**	/	/	/
PO_ISCB	1	5.35	160.60	0.70	**0.40**	0.24	0.53	8.13
PO_ILNCD	3	5.40 ± 0.11	127.17 ± 11.70	3.10 ± 1.50	**2.27 ± 1.16**	0.24 ± 0.14	1.87 ± 0.99	7.88 ± 3.97
PO_ILNCB	2	5.70 ± 0.15	148.56 ± 1.37	5.45 ± 0.78	**4.82 ± 0.68**	0.49 ± 0.01	0.86 ± 0.56	8.00 ± 4.99
PO_ILCD	0	/	/	/	**/**	/	/	/
PO_ILCB	4	5.76 ± 0.51	286.67 ± 6.84	110.42 ± 40.73	**106.08 ± 40.65**	8.85 ± 2.58	0.94 ± 1.32	6.31 ± 5.19
CC_SNCD	12	5.62 ± 0.21	133.02 ± 4.83	1.58 ± 0.70	**1.44 ± 0.72**	0.19 ± 0.08	1.90 ± 1.49	0.51 ± 0.28
CC_SNCB	11	5.50 ± 0.31	132.49 ± 6.66	1.35 ± 0.55	**1.16 ± 0.54**	0.20 ± 0.13	2.46 ± 1.60	0.60 ± 0.52
CC_SCD	14	7.85 ± 0.72	239.52 ± 27.84	25.84 ± 12.69	**22.34 ± 11.38**	2.85 ± 1.25	1.16 ± 1.06	0.58 ± 0.51
CC_SCB	15	6.70 ± 0.30	291.66 ± 25.11	53.06 ± 25.14	**48.39 ± 23.37**	5.77 ± 2.26	1.27 ± 0.81	0.67 ± 0.56
CC_LNCD	0	/	/	/	**/**	/	/	/
CC_LNCB	0	/	/	/	**/**	/	/	/
CC_LCD	0	/	/	/	**/**	/	/	/
CC_LCB	0	/	/	/	**/**	/	/	/

## Data Availability

Data is available upon request to the authors.

## Appendix A

The percentages of each ingredient used in inoculation recipes from the literature are presented here. The GrAB project team used the following averaged percentages: 10 wt% of *Pleurotus ostreatus* spawn, 14 wt% of substrate, and 76 wt% of water [79]. Jones et al., 2018, soaked the substrate in Type 1 Milli-Q^®^ water and sterilized the mixture before adding 25 wt% of *Trametes versicolor* spawn, while explaining that lower percentages of spawn slowed mycelial growth and increased risks of contamination [18]. Elsacker et al., 2019, used the following recipe in weight percentages: 10 wt% of *Trametes versicolor* spawn, 20 wt% of substrate (chipped, sieved and sterilize), and 70 wt% of sterile demineralized water [9]. The LIWAS project used the following recipe: 6 wt% of *Hypsizygus ulmarius* mycelium spawn, 34.5 wt% of substrate, and 59.5 wt% of water [5]. In comparison with other projects, these last percentages produced mixed mycelium growth results, likely due to the low amounts of mycelium spawn provided [18].

## Appendix B

Details about sieving non-spherical objects and the sieving procedure conducted are described here. Due to the high aspect ratio between the length and width and/or thickness of the chipped material, the use of sieves is not as straightforward as with more spherical objects. The amount of material passing through the sieve depends on the amount of material placed on the sieve, the movement produced for sieving, and the sieving time. If too much material is placed on the sieve, particles are more likely to be arranged in a position perpendicular to the sieve holes (vertically). The horizontal sieving technique was chosen to limit the vertical repositioning of the particles after being positioned on a sieve. The speed of the circular sieve movement can also cause the particles to reposition vertically. Therefore, these three variables were kept constant throughout the sieving of the chipped straw.

The sieving process for the 5.7 mm sieve consisted of placing 140 g of chipped material on the 5.7 mm sieve and making 10 horizontal circles with the sieve for 8 s in a clockwise direction, then in an anti-clockwise direction. The sieving process for the 1.5 mm sieve consisted of placing 70 g of chipped material on the 1.5 mm sieve and making 5 horizontal circles with the sieve for 4 s in a clockwise direction, then in an anti-clockwise direction.

## Appendix C

The details of the procedure performed to quantify the particle sizes of both mixture are presented here. First, particles were positioned on a light table while making sure that they did not touch each other. If particles were touching each other, the algorithm would recognize them as one, and resulting dimensions would be false. A picture of these particles was taken from a top view with a Sony alpha 7R II camera (Sony, New York City, NY, USA) mounted on a tripod. The following settings were used: f/4, 1/100 s, ISO-100 at a focal length of 35 mm. The 35 mm focal length was used to reduce lens distortion. The picture was then opened in Adobe Lightroom (version 6.10.1, Adobe^TM^, San Jose, CA, USA) to crop, increase contrast, and correct the lens distortion. The adjusted pictures were then imported into a Python script (version 3.7, Python Software Foundation, Wilmington, DE, USA). The script turned the image into a binary image (e.g., only composed of black or white pixels), labeled each individual item, and calculated the number of objects and the area, perimeter, eccentricity, major axis length, and minor axis length of each item. A ruler integrated in the light table was used to scale the image by converting the number of pixels from the picture to mm.

## Appendix D

The pasteurization process can be divided into the following steps: first, the substrate mixture (S or L) is placed in a large steamer with a rotating arm in its center. Then, the required amount of water is added to the mixture based on the necessary percentages from the inoculation recipe (detailed in Section 2.3). The steamer is then closed and both mixing and steaming are started. The steamer is kept on for one hour at a temperature of 64–65 °C to allow proper pasteurization. A thermal blanket is placed around the mixer to retain the heat. The mixture is then allowed to cool for one hour until it reaches a temperature of 27 °C. To speed up the cooling, the mixer is kept on and the fan of the steamer blows filtered outdoor air through the mixture. To enhance the pasteurization process, water is introduced before pasteurizing the substrate. However, the pasteurization process also brings moisture in. To quantify the quantity of water added to the mixture during the pasteurization process, the team ran the steamer without any substrate. The steamer produced approximatively 5.90 L of water, which represented 13.40% of the water in our inoculation recipe.

## Appendix E

The inoculation procedure of both batches and summary of the quantity of materials grown are described here. Mixture L of the first batch was inoculated as follows: first, 9303.3 g of sterilized dry substrate was placed inside a sterilized drum mixer. With the drum mixer on (i.e., rotating), 28 L of distilled water was slowly poured inside the mixer. The mixer was left running to mix the substrate and water for 10 min. Then, 4134.8 g of *Pleurotus ostreatus* grain spawn was added. The whole mixture was mixed for an additional 30 min. The molds were sterilized with 70% isopropyl alcohol. After 30 min of mixing, the mixture was placed and compacted into the sterilized molds. The mixer was kept running while the team filled the molds to prevent the mixture from sitting still for too long. An acrylic disk was used for pushing by hand as hard as possible to compact the mixture multiple times during the filling process. For the 17 cm tall PVC tubes, the mixture was compacted twice: when the mixture filled half of the tube height and when it filled the entire height. For the 34 cm tall PVC tubes, the mixture was compacted four times (i.e., at each quarter of the height). Once the molds were filled, specimens for compression testing were covered with a layer of non-woven housewrap (Everbilt, Home Depot, USA) to maintain constant humidity, reduce the exposure to contaminants, and limit mycelium growth on the outer surfaces. In mycelium materials, the surfaces exposed to ambient air display increased mycelial growth. This phenomenon impacts mechanical behavior as shown in a study about jacketing the materials [44]. The bias emerging from increased mycelium growth on external surface was reduced in our study by compacting mixtures in the molds and growing all specimens in PVC tubes with top and bottom surfaces covered with housewrap. Specimens for bending testing were placed inside a ridge-like tent. The ridge-like tent was made of non-woven housewrap and clear plastic, to ensure mycelium growth was visible throughout the growth period. All covered molds were finally placed inside a sterilized large tent in order to lower the risks of contamination. An opaque tarp covered the large tents containing the specimens during the growth process to block sunlight, which is a stimulus for the formation of fruiting bodies. Filtered air flowed into this tent throughout the mycelial growth period. The same process was performed for mixture S with the following quantities: 11,841.3 g of sterilized dry substrate, 35.5 L of distilled water, and 5262.8 g of *Pleurotus ostreatus* grain spawn. The leftover inoculated mixtures, S and L, filled four additional rectangular molds each. The filled molds were then sealed with a layer of non-woven housewrap.

For the second batch, water was added before the pasteurization process and the mycelium spawn after it. Once the mixture had cooled to 27 °C, the mycelium spawn was added. After 5 min of mixing, the inoculated mixture was placed inside opaque trash bags to be transported to the growth facility (redhouse studio’s warehouse in Cleveland, OH, USA). The temperature of the inoculated mixture should remain constant to avoid early growth and contaminants. Therefore, bags were only half filled with an average of 13.4 kg of inoculated mixture. These bags were then spread on a grid to reduce the heat produced by this biomass. Due to the time needed to fill all the molds, mixture S was placed in the molds on the day following inoculation, whereas mixture L was placed two days after inoculation. After being sterilized with 70% ethanol, the molds were filled with the inoculated mixture and pressed to achieve the desired heights akin to the previous batch. Finally, the filled molds were weighed and placed in their respective growth environments. The growth environments used for the first batch were reused for the second batch. In the first batch, the closed PVC tubes were set on a planar surface to maintain a flat surface on the specimens’ bottom sides. However, this configuration did not let the specimens fully breathe during the growth period resulting in high humidity of the lower parts of the specimens and the presence of higher rates of contamination. Therefore, for the second batch, the filled PVC molds were placed on a metal grid to let them breathe. The leftover inoculated mixtures, S and L, filled eight additional rectangular molds each. Similar to the first batch, a layer of non-woven housewrap was used to cover these filled molds.

A total of 120 specimens were grown to evaluate the effects of the following variables on the compressive properties of mycelium-based composites: fungal species (*Pleurotus ostreatus* and *Coprinus comatus*), substrate particle sizes (with or without micro-particles), and post-growth treatments (dried, baked, compacted then dried, and compacted then baked). A total of 40 specimens were grown for the first batch with *Pleurotus ostreatus* to achieve a sample size of five. Due to increased availability of materials, space, time, and levels of contaminations observed in the first batch, the sample size was increased to 10 (80 specimens total) for the second batch grown with *Coprinus comatus*. For bending testing, the effects of the same variables were evaluated with a total of 148 specimens. The sample sizes used were 6 for the first batch (48 specimens) and 12 or 15 for the second batch (100 specimens), depending on the number of molds and material available.

## Appendix F

Additional details about the growth data collected and removal of contaminated parts of the materials are discussed here. At the end of the growth process, each specimen was weighed and photographed, and its dimensions were measured. The contamination and fruiting bodies were removed throughout the drying process. In addition, pictures were taken before and after the removal of both fruiting bodies and contaminated material. Fruiting bodies and contamination were weighed as they were removed. Contaminants were seen growing on fruiting bodies. Since all specimens were kept in the same tent, keeping contaminated specimens would increase the risk of contamination of the non-contaminated specimens. However, as mycelial growth requires oxygen, mycelium growth was less abundant inside the specimens than on their surface. Therefore, cutting off contaminated parts exposed the interior of the specimens which contained substrate mixture with lower mycelial growth. This exposure promoted contaminant growth on drying panels. In this regard, the specimens were either left untouched, partly cut off, or fully disposed of, based on the amount of contamination.

## Appendix G

Additional information about the application of post-growth treatments is presented here. Post-growth treatments (drying, baking, compacting then drying, or compacting then baking) were applied to the fully grown specimens in two locations: the Biodesign lab at the University of Akron and redhouse studio’s warehouse where the specimens were grown. The relative humidity and presence of contaminants was lower in the Biodesign lab than at the warehouse. For instance, no growth of contaminants was observed on the panels brought to the Biodesign lab, whereas some contaminants started to grow on the panels kept at redhouse studio’s warehouse throughout the post-growth treatment procedure. Therefore, non-compacted panels were baked or dried at the University of Akron to reduce the exposure to humid air and contaminants from the warehouse. The rest of the specimens were compacted, and dried or baked at redhouse studio’s facility due to equipment availability. With the post-growth treatments applied, materials were moved to the Biodesign lab at the University of Akron for storage and mechanical testing.

To compact materials from the first batch, layers of aluminum foil were placed between the specimen and the metal plates to prevent the myceliated specimens from sticking to the metal plates. After the baking or drying process, some of the aluminum foil remained stuck to the myceliated specimens and metal plates. Separating the specimens from the aluminum foil and metal plates damaged some specimens. For the second batch, the aluminum foil was replaced with a polyester film, called mylar or biaxially-oriented polyethylene terephthalate (BoPET). In addition to its resistance to temperatures up to 100 °C, this material did not stick to the myceliated specimens. Once the compaction load was removed, the specimen recovered thickness. Therefore, the panel specimens (i.e., grown for bending testing) were kept compacted with metallic clamps. These clamps were only removed after the full drying or baking process. To avoid lateral expansion upon compaction, cylindrical specimens (i.e., grown for compression testing) were compacted inside of their respective growth environments (i.e., PVC pipes). However, clamps could not be placed until the specimens were taken out of the tubes. The load was kept on the specimen for a minimum of 5 min. Then, the specimens were removed from the pipes and kept compacted with clamps to maintain the desired thickness.

## Appendix H

Additional details about the calculation of Young’s modulus and the analysis of the bending test data are described here. Throughout compression testing, the cross-sectional area of the specimen increases. To calculate the compressive stress, ASTM D2166/D2166M-13 states that the cross-sectional area of the specimen used in the equation should account for specimen deformation throughout the test [55]. The ASTM standard contains a formula to estimate the cross-sectional area of a specimen for a given load. This formula was used for one of the specimens that had an initial cross-sectional area of 42.19 cm^2^ before the test. The formula estimated a cross-sectional of 168.76 cm^2^ after 75% deformation. However, the measured cross-sectional area was 59.58 cm^2^ after 75% deformation. Therefore, this formula was not used in the calculations of compressive stresses.

In addition to the load-deflection data, the modulus of elasticity, maximum load, and flexure extension at maximum load were collected from the Instron. However, ASTM D1037-12 contains a specific formula to calculate the modulus of elasticity. The moduli from the Instron reading and the ASTM calculation were slightly different. The modulus calculated by the Instron was higher in most cases. Since the calculation used to find the modulus by the Instron is not known by the authors, the modulus of elasticity calculated from the ASTM standard was kept for data comparison. Following the standard, the slope of the straight-line portion of the load–deflection curve was calculated with a linear regression of the load–deflection curve between 10% and 40% of the maximum load.

The figure below presents how the fracture offset from the center and fracture angle were measured for the analysis of the specimens after the bending tests.

## Appendix I

The evolution of mycelium and contaminant growth in the first batch is briefly described here. During the growth process, the state of the panels placed was visually assessed in a see-through enclosure. Environments were kept closed to limit contamination. Eleven days after inoculation, small amounts of mycelial growth were observed on the specimens. However, grey and green spots, likely contaminants, were seen. Twenty-one days after inoculation, mycelium formed a thin mat over most of the visible specimens’ surfaces. The grey and green spots were still present. Forty-six days after inoculation, the mycelium formed a thick mat over the specimens, and the growth environments were opened for thorough assessment.

Further information about the height shrinkage and weight reduction of both cylindrical and rectangular specimen types is presented here. The shrinkage of the specimens grown for compression testing (i.e., in PVC pipes) was calculated by comparing the weight and dimensions of all specimens at the following stages: (1) inoculation, (2) beginning of drying (i.e., when growth environments were opened at the end of the growth period), (3) before cutting (i.e., specimens had to be cut to the sizes required for testing), (4) before the application of post-growth treatments, (5) before baking or drying, and (6) before mechanical testing (Table A1). To limit the growth of the contaminants after the mycelium growth period, contaminated parts of the specimens were removed from the grown materials. These parts do not possess mycelial growth. As such, they should not be used for the mechanical tests since they would generate invalid data. Removing these pieces jeopardized the comparison of the height and weight measurements. The width was not affected as the contaminated pieces were mostly slices at the top and bottom of the specimens. Therefore, upper or lower portions of the specimens were removed. In this regard, the weight evolution was analyzed for the specimens, with less than 10 g of contamination removed throughout the entire process.

**Table A1:** This table shows the evolution of the weight, height, and diameter from inoculation to the beginning of compression testing. Specimens were cut between steps 3 and 4. The weight was therefore kept between these two steps to ensure the comparison was continuous. The height was reset to 100% once the specimens were cut.

Step of the Process	Weight Compared to Initial (%)	Height Compared to Initial (%)		Diameter Compared to Initial (%)
	All Samples	Uncompacted	Compacted	All Samples
1. Inoculation	100.00	100.00		100.00
2. Beginning of drying	95.37	100.00		100.00
3. Before cutting	50.95	95.00		/
4. Before post-growth treat.	50.95	100.00	100.00	96.76
5. Before baking or drying	51.90	/	50.14	/
6. Before mechanical test	21.91	99.56	49.54	96.48

The weight and dimensions of all specimens grown for bending testing were measured at the following steps: (1) inoculation, (2) beginning of drying, (3) before the application of post-growth treatments, (5) before baking or drying, and (6) before cutting to required sizes for testing (Table A2). In contrast to cylindrical specimens, the post-growth treatments were applied to the specimens before cutting them to fit testing requirements for practicability. Each grown specimen was cut into six test specimens directly before the bending testing. All specimens grown for bending contained some contaminants. The weight of contamination manually removed during step 2 accounted for 28.84% of the initial weight of the specimens at inoculation. However, the uncontaminated parts of the specimens had to be trimmed further to fit the size requirements for testing. In total, 42.78% of the initial weight was removed and could not be used for testing. Therefore, tracking the evolution of these measurements was more challenging. The data were divided into two sets to facilitate the analysis: all specimens, and only those on which less than 10% of contamination was removed throughout the entire process.

**Table A2:** This table shows the evolution of the weight, height, width, and length for the specimens grown for bending. Uncompacted (NC) and compacted (C) specimens were analyzed separately since compacted specimens were constrained in metal frames used for compaction.

Step of the Process	Weight Compared to Initial (%)		Height Compared to Initial (%)		Width Compared to Initial (%)		Length Compared to Initial (%)	
	NC	C	NC	C	NC	C	NC	C
Specimens with less than 10% contamination removed								
1. Inoculation	100.00	100.00	100.00	100.00	100.00	100.00	100.00	100.00
2. Beginning of drying	78.82	85.21	111.90	99.93	97.65	98.40	98.50	99.13
3. Before post-growth treat.	17.00	50.13	91.07	91.07	94.93	/	96.50	/
4. Before baking or drying	/	/	/	/	/	/	/	/
5. Before cutting	/	15.17	87.14	37.32	/	92.35	/	93.05
**All specimens**								
1. Inoculation	100.00	100.00	100.00	100.00	100.00	100.00	100.00	100.00
2. Beginning of drying	81.17	84.27	102.32	99.93	98.25	98.40	99.00	99.13
3. Before post-growth treat.	37.65	50.13	91.96	91.07	94.93	/	96.50	/
4. Before baking or drying	/	/	/	/	/	/	/	/
5. Before cutting	/	15.17	90.71	37.32	95.07	92.35	/	93.05
